# Indium Imidazo[4,5,-*b*]porphyrins as Photocatalysts for Oxidation of Sulfides

**DOI:** 10.3390/molecules30040864

**Published:** 2025-02-13

**Authors:** Inna A. Abdulaeva, Mikhail A. Filatov, Azhar Kechiche, Alla Bessmertnykh-Lemeune

**Affiliations:** 1 Institut de Chimie Moléculaire de l’Université de Bourgogne, UMR 6302 CNRS, Université de Bourgogne, 9 Avenue Alain Savary, BP 47870, 21078 Dijon, CEDEX, France; inna.a.abdulaeva@gmail.com; 2 Frumkin Institute of Physical Chemistry and Electrochemistry, Russian Academy of Sciences, Leninsky Pr. 31-4, 119071 Moscow, Russia; 3 School of Chemical and Biopharmaceutical Sciences, Technological University Dublin, City Campus, Grangegorman, Dublin 7, D07 ADY7 Dublin, Ireland; 4 ENS de Lyon, CNRS, LCH, UMR 5182, 69342 Lyon, CEDEX 07, France; azhar.kechiche@ens-lyon.fr

**Keywords:** porphyrin, sulfide, phosphonate, photooxidation, hybrid materials

## Abstract

Over the past two decades, the application of photocatalytic reactions in organic synthesis has increased remarkably. Porphyrins, renowned for their exceptional photophysical properties, photostability, and prevalence in natural catalytic processes, are attracting significant attention as promising photocatalysts for reactions proceeding through energy transfer and one-electron transfer. In this work, we synthesized the indium(III) complex of 2-[4-(diethoxyphosphoryl)phenyl]-1*H*-imidazo[4,5-*b*]-5,10,15,20-tetramesitylporphyrin (InTMPIP) and explored its application as a photocatalyst for the oxidation of sulfides by dioxygen or air. Complex InTMPIP was found to generate singlet oxygen with quantum yield of 0.92 (toluene) and enables efficient photooxidation of sulfides to sulfoxides by dioxygen in “green” acetonitrile/water (4:1 *v*/*v*) or methanol/chloroform (2:1 *v*/*v*) solvent mixtures with almost quantitative yield. Furthermore, InTMPIP was grafted onto hydrated mesoporous titania and materials InTMPIP/TiO_2_-1 and InTMPIP/TiO_2_-2 with different In/Ti ratios were obtained and investigated. The composition and structure of the materials were studied using a combination of elemental analysis, various spectroscopic methods, gas adsorption measurements, and SEM imaging. Finally, the photocatalytic efficiency of InTMPIP/TiO_2_-2 was explored in aerobic photooxidation of sulfides. The heterogenized complex enables selective synthesis of sulfoxides under “green” conditions; however, it is prone to leaching into the solution when irradiated with both blue and red LEDs.

## 1. Introduction

The advancement of various fields in physics, chemistry, and biochemistry depends significantly on our ability to conjugate diverse functional molecules via covalent linkages. This enables the synthesis of sophisticated modular compounds with specific properties or derivatives that combine the characteristics of individual residues within a molecular system with fixed spatial arrangements. The widespread popularity of “click chemistry”, which enables the coupling of complementary molecules bearing, for instance, azide and alkyne groups, serves as an excellent example of the importance of these synthetic methods. Beyond the “click” approach, organic chemistry offers numerous alternatives, including oxidative coupling, functional group modifications (e.g., ester or amide formation reactions), and methodologies involving the installation of linkers such as acetylene, 1,4-phenylene, 1,4′-biphenylene, or heteroaromatic spacers. Among these, fused imidazole spacers have garnered significant interest, particularly in the synthesis of metal complexes with versatile nitrogen-containing ligands, such as 1,10-phenanthrolines and porphyrins [1,2,3,4,5].

**Figure 1:**
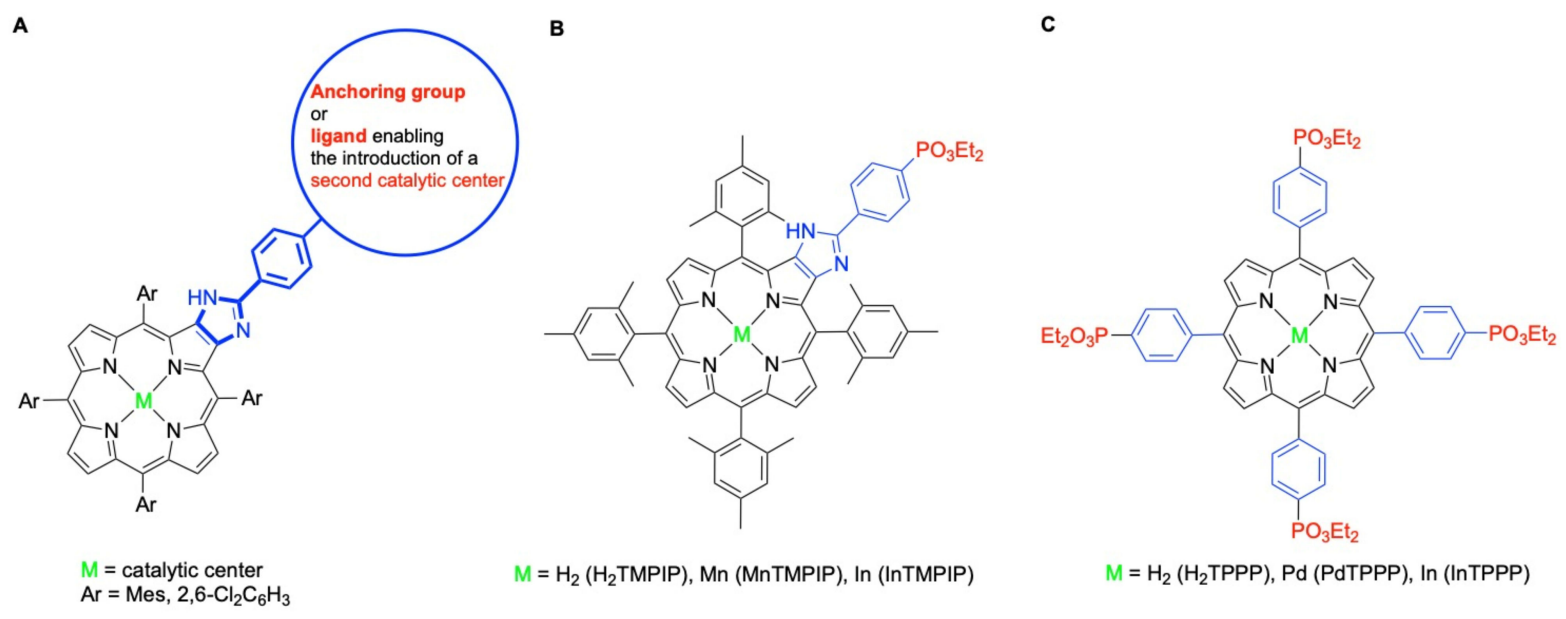
(**A**) Conjugation of *meso*-tetraarylporphyrins and functional residues via a fused imidazole linker. (**B**) The structure of imidazo[4,5-*b*]porphyrin InTMPIP, investigated in this work, and reference compounds. (**C**) The structure of reference phosphonate-substituted porphyrins MTPPP.

In porphyrin chemistry, this approach was pioneered by Crossley’s group, which developed the synthesis of 2-phenyl-substituted imidazo[4,5-*b*]porphyrins (Figure 1A) [6,7,8,9]. The challenging synthesis of such derivatives, which involves a photochemical step, likely explains why this conjugation methodology has only recently attracted broad attention. These recent studies demonstrated that this synthetic route is both reproducible and scalable, irrespective of the nature of the *meso*-aryl substituents on the tetrapyrrolic macrocycle [10,11,12,13]. Moreover, imidazo[4,5-*b*]porphyrins unsubstituted at the 2-position of the imidazole ring were prepared and widely investigated as precursors of porphyrin–*N*-heterocyclic carbene (porphyrin–NHC) conjugates by Richeter’s group [14,15,16].

Our interest in these compounds stems from our studies on the application of porphyrins as catalysts [17]. Recently, we demonstrated that this methodology is useful for heterogenization of porphyrins belonging to the second generation [18,19,20] of catalysts (complexes with *ortho*-substituted and electron deficient H_2_TPP derivatives) [21]. Obtained imidazo[4,5-*b*]porphyrins bearing the *meso*-tetraarylporphyrin residue and an additional functional group (Figure 1A) hold potential for the development of tandem, metallaphotoredox, and reusable catalysts due to the acid–base properties of the imidazole linker and the possibility of introducing different anchoring groups or a second catalytic center at the 2-position of the imidazole ring. This functionalization minimally perturbs the electronic structure of the porphyrin’s catalytic core due to the low degree of conjugation between the imidazole ring and the aromatic macrocycle, which is advantageous for catalyst development. In our previous work, the manganese complex MnTMPIP (Figure 1B) was investigated as a catalyst under homogeneous conditions and then grafted onto hydrated mesoporous titania supports via the phosphonate anchoring group [22]. We also demonstrated that the resulting mesoporous hybrid material could serve as an efficient and reusable catalyst for oxidation reactions.

In this work, we investigated imidazo[4,5-*b*]porphyrins and materials derived from these compounds in photocatalysis, a field that has recently emerged as one of the primary focuses in organic synthesis [23,24,25,26]. Specifically, we synthesized the indium(III) complex InTMPIP and explored its application as a photocatalyst (PC) in oxidation reactions where dioxygen or air is used as the terminal oxidant.

Oxidation reactions are key processes in the chemical industry, which is based on converting fossil fuel-derived feedstocks into value-added compounds [27,28]. These transformations are especially challenging and environmentally burdensome because they often require stoichiometric amounts of toxic and hazardous reagents, such as oxo-metal oxidants or peracids, and tend to proceed with low selectivity [29]. The development of oxidation reactions employing dioxygen in non-toxic solvents is therefore vital from both economic and environmental perspectives [30,31,32]. Singlet oxygen, which can be generated from dioxygen in the presence of photosensitizers (PSs; commonly referred to as photocatalysts (PCs) in organic synthesis) and visible light, has long been recognized as a promising oxidant for a wide range of substrates. Various types of dyes have been reported to efficiently generate this reactive oxygen species [33,34]. Among metalloporphyrins, palladium(II) complexes are commonly used as PSs due to their superior efficiency in generating singlet oxygen and their higher photostability compared to free-base porphyrins and Zn(II) complexes [35,36]. Recently, indium(III) complexes, known as efficient PSs for photodynamic therapy [37,38], have emerged as an alternative to palladium (II) porphyrins in photocatalysis [39]. Despite indium being a precious metal, it is approximately 40 times less expensive than palladium and the insertion of In(III) ions into tetrapyrrolic macrocycles generally proceeds under mild conditions. Moreover, In(III) complexes structurally differ from Pd(II) porphyrins due to the presence of an axial ligand, which could potentially participate in the photocatalytic transformation. While the comparison of photocatalytic properties between In(III) and Pd(II) porphyrins remains limited [17], the available data suggest that these complexes are more efficient and photostable than free-base porphyrins and Zn(II) complexes [17,39]. To our knowledge, In(III) and Pd(II) complexes of imidazo[4,5-*b*]porphyrins have not been previously reported. Thus, we focus our work on the synthesis of the In(III) complexes of imidazoporphyrins with the goal of developing practical oxidation processes. Using the conjugation of *meso*-tetramesitylporphyrin and 2-[4-(diethoxyphosphoryl)phenyl] residues via a fused imidazole linker, followed by the insertion of In(III) in its tetrapyrrolic macrocycle, we synthesized the indium(III) imidazo[4,5-*b*]porphyrin InTMPIP (Figure 1B). We demonstrated that this complex generates singlet oxygen in toluene with quantum yield near unity. InTMPIP was further successfully used for the selective oxidation of sulfides to sulfoxides. The introduction of a diethoxyphosphoryl group on the imidazo[4,5-*b*]porphyrin core significantly increased the solubility of InTMPIP in various organic solvents and aqueous mixtures, as previously reported [17] for *meso*-tetraphenylporphyrins (MTPPP, M = Pd(II), In(III), Figure 1C). Due to good solubility of InTMPIP, homogeneous photooxidation of sulfides can be performed not only in a CHCl_3_/MeOH mixture but also in a more environmentally friendly MeCN/H_2_O solvent mixture. We also investigated covalent grafting of this PS onto a mesoporous titania support to prepare a reusable photocatalyst, aiming to improve the sustainability of the oxidation process. This novel strategy for catalyst immobilization, developed by our group [22,40], enables the preparation of reusable catalysts for cross-coupling reactions and sulfoxidation using a dioxygen/isobutyraldehyde (IBA) system. Material prepared by a sol–gel process from InTMPIP efficiently generated singlet oxygen and enabled selective synthesis of sulfoxides in MeOH, but the heterogenized complex was found to be prone to leaching when irradiated with both blue and red LEDs.

## 2. Results and Discussion

### 2.1. Synthesis of InTMPIP

2-[4-(Diethoxyphosphoryl)phenyl]-1*H*-imidazo[4,5-*b*]-5,10,15,20-tetramesitylpor-phyrin (H_2_TMPIP) was prepared on a hundred-milligram scale using a procedure previously reported by us [21]. For the insertion of In(III) ions into the tetrapyrrolic macrocycle, indium(III) chloride was used as the metal source, and the reaction was carried out in a refluxing acetic acid with sodium acetate added to reduce the acidity of the reaction mixture. The target complex was isolated by column chromatography in 95% yield and characterized using spectroscopic techniques and high-resolution mass spectrometry (see the Electronic Supplementary Information).

Consistent with our earlier findings on phosphonate-substituted porphyrins [17], InTMPIP exhibited high solubility in a range of organic solvents, including chlorinated solvents, MeCN, toluene, tetrahydrofuran, and dimethylformamide, at concentrations exceeding 0.5 mM. However, its solubility in alcohols was significantly limited. Interestingly, while solid InTMPIP was insoluble in an MeCN/H_2_O (4:1 *v*/*v*) mixture, a 0.02 mM solution was successfully prepared by dissolving InTMPIP in pure MeCN and then adding the calculated amount of water. This behavior is likely due to axial coordination of acetonitrile molecules by InTMPIP, leading to the formation of a stable complex (InTMPIP·MeCN). Unfortunately, attempts to grow single crystals of InTMPIP from acetonitrile to confirm this hypothesis were unsuccessful. Notably, the In(III) complex of the commercially available *meso*-tetraphenylporphyrin (InTPP) is insoluble in alcohols, MeCN and aqueous solvent mixtures; this significantly limits its potential in homogeneous photocatalytic reactions.

### 2.2. Optical Properties of H_2_TMPIP and InTMPIP, and Singlet Oxygen Generation

**Figure 2:**
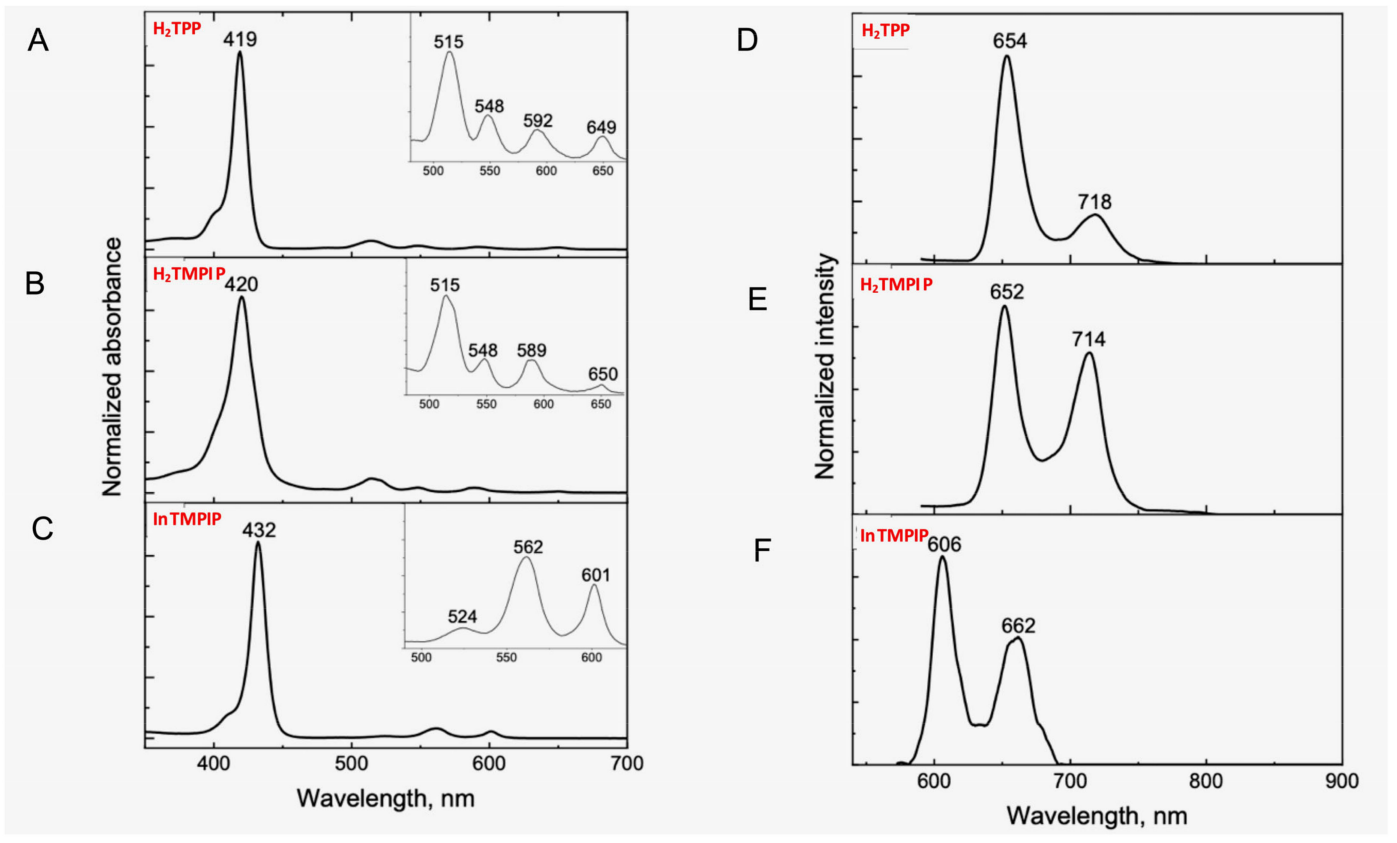
UV-vis spectra (panels **A**–**C**) and emission spectra (panels **D**–**F**; *λ*_exc_ = 520 nm) of H_2_TMPIP (**B**,**E**), InTMPIP (**C**,**F**), and reference compound H_2_TPP (**A**,**D**) in toluene.

The electronic absorption spectra of H_2_TMPIP and InTMPIP in CHCl_3_ and toluene closely resemble those of H_2_TPP and InTPP, respectively, with a prominent Soret band in the near-UV–visible region (380–450 nm) and Q bands in the range of 500–670 nm (Figure 2 and Table S1). A red-shift of the absorption maxima induced by insertion of the In(III) ion was observed in line with previous reports on imidazolium-substituted porphyrins [41].

Upon excitation at the Q bands, both the free-base porphyrin H_2_TMPIP and the In(III) complex InTMPIP exhibit a split fluorescence band, corresponding to the Q(0,0) and Q(0,1) transitions (Figure 2). The emission maxima of the In(III) complex are blue-shifted relative to those of the parent free-base porphyrin. Furthermore, the fluorescence quantum yield (*Φ*_f_) decreases significantly from 0.101 to 0.015 upon metal ion insertion into the imidazoporphyrin core. This decrease is expected, as heavy atoms like indium promote intersystem crossing due to strong spin–orbit coupling caused by the heavy atom [42,43]. Notably, the emission spectra of H2TMPIP and InTMPIP are similar to those of H2TPP and InTPP, respectively (Figure 2). Moreover, the UV-vis and emission spectra of phosphonated porphyrins MTPPP (M = H_2_, In(III)), in which four phosphonate substituents are separated from the tetrapyrrolic macrocycle by 1,4-phenylene linkers, also resemble those of H_2_TMPIP and InTMPIP (Figure S1). These spectral data support the hypothesis that the introduction of the phosphonate anchoring group via the fused imidazole linker does not significantly perturb the frontier molecular orbitals of H_2_TPP and its In(III) complex.

Based on *Φ*_f_ values of H_2_TMPIP and InTMPIP, the free-base porphyrin H_2_TMPIP appears to be a less promising PS compared to its indium complex, owing to the heavy atom effect observed in the latter compound. To validate this conclusion, the efficiency of singlet oxygen generation by H_2_TMPIP and InTMPIP was evaluated. Singlet oxygen quantum yields (*Φ*_Δ_) were measured in toluene using an indirect method with 1,9-dimethylanthracene (DMA) as a chemical trap. Upon irradiation of air-saturated solutions containing each PS at 514 nm, DMA selectively reacts with singlet oxygen to form the corresponding endoperoxide. Neither H_2_TMPIP nor InTMPIP samples exhibited any change in absorption during the course of irradiation (Figure S2). The decrease in DMA absorbance over time was linear for both compounds (Figure S2, inset), enabling the determination of *Φ*_Δ_ by comparison with the reference compound—*meso*-tetraphenylporphyrin (H_2_TPP). The InTMPIP complex generated singlet oxygen with *Φ*_Δ_ of 0.92, which is comparable to those of the well-known PdTPP and its phosphonated analogue PdTPPP [17]. In contrast, H_2_TMPIP exhibited *Φ*_Δ_ of 0.65, similar to that of H_2_TPP [44]. Notably, the *Φ*_Δ_ of InTMPIP is significantly higher than those of conventional organic photosensitizers, such as Methylene Blue (0.5) and Rose Bengal (0.8) [34]. These results suggest that the photophysical characteristics of InTMPIP are highly favorable for applications in the photooxidation of organic compounds using dioxygen as a terminal oxidant.

### 2.3. Homogeneous Photooxidation of Sulfides

Metalloporphyrins have been extensively studied for the oxidation of alkanes, alkenes, alcohols, aldehydes, and sulfides. In this work, the oxidation of sulfides to sulfoxides was chosen as a model reaction due to its significant societal relevance [45,46,47,48,49,50] and the inherent challenges of achieving selective sulfide-to-sulfoxide transformation [51,52,53,54]. For instance, precise control of the reaction time is essential to achieve selectivity when using the dioxygen/IBA system in the presence of imidazoporphyrin MnTMPIP, as rapid overoxidation of the target sulfoxide to sulfone occurs once the sulfide is fully converted [22]. The sulfoxidation by dioxygen can also be carried out without a sacrificial reagent (IBA) by utilizing a photocatalyst under visible light irradiation [50,55,56]. When porphyrins are used as PCs, the reaction predominantly proceeds via energy transfer (EnT) from the PC to dioxygen, generating singlet oxygen that subsequently reacts with sulfides [17]. However, the involvement of alternative mechanisms, proceeding via one-electron transfer, cannot be entirely excluded, particularly in the oxidation of aryl sulfides [57,58,59,60]. Previous studies have shown that the photooxidation of sulfides by singlet oxygen is significantly enhanced in the presence of protic additives, such as alcohols and phenols [61,62]. Solvent mixtures like CHCl_3_/MeOH [63] or MeCN/H_2_O [64] have been found to be particularly suitable for the preparative synthesis of sulfoxides. Therefore, in the current study, we explored the photooxidation in both solvent mixtures.

Initially, the reaction was studied in a MeCN/H_2_O mixture (4:1 *v*/*v*) under a pure oxygen atmosphere (0.75 L balloon) while being irradiated with a 425 nm LED (18 W) in an EvoluChem Photoredox Box. The photooxidation of thioanisole was completed in less than 1 h using 0.05 mol% of the PC, yielding the target sulfoxide with high selectivity (Table 1, entry 1). In contrast to chemical oxidation with the dioxygen/IBA system [22], overoxidation occurred at a much slower rate, with only trace amounts of sulfone detected even after 2 h of irradiation. The essential role of both the PC and visible light irradiation was confirmed in control experiments conducted under identical conditions but without either the photocatalyst or LED irradiation, where no product formation was observed. Remarkably, when the PC loading was reduced by a factor of 10, phenyl methyl sulfoxide was still obtained after 1 h of irradiation (Table 1, entry 2).

As shown in Table 1, under these conditions, most aryl methyl sulfides were converted to sulfoxides with near-quantitative yields, and less than 2% of overoxidation products were observed in most of the reactions. The oxidation of electron-rich 4-methoxythioanisole (entry 8) as well as electron-deficient 4-chlorophenyl and 4-cyanophenyl methyl sulfides (entries 10 and 14) was completed within 1 h. Remarkably, bulky *ortho*-bromothioanisole was oxidized in 1 h (entry 15), despite previous reports indicating that *ortho*-substitution typically significantly reduces the photooxidation rate [17,64]. It is important to note that this work was primarily focused on developing a practical synthetic procedure, and detailed kinetic studies were beyond its scope.

4-Nitrothioanisole, a sulfide generally inert in EnT reactions [64], exhibited low reactivity, achieving only 40% conversion after 20 h of irradiation (entry 18). Notably, increasing the PC loading to 0.05 mol% resulted in only a slight acceleration of the reaction (entry 19), likely due to an inefficient light absorption by the PC molecules under these conditions. As a result, the target product was obtained in a 98% yield after 20 h of irradiation. Similarly, the oxidation of 4-aminophenyl methyl sulfide, which contains an amino group prone to side reactions via electron transfer, proceeded slowly, with 77% conversion observed after 10 h of irradiation (entry 22). Fortunately, diphenyl sulfide, which is also resistant to photooxidation by singlet oxygen due to its low nucleophilicity and steric hindrance [65], was efficiently oxidized after 6 h of irradiation (entry 24).

InTMPIP also demonstrated high efficiency in the oxidation of dialkyl sulfides. Dibutyl sulfide reacted smoothly under these conditions, affording the sulfoxide in 95% yield within 1 h (entry 27). Cyclic thian-4-one, known for its lower reactivity compared to acyclic derivatives [17] was nonetheless quantitatively oxidized within 1 h (entry 29).

When the MeCN/H_2_O solvent mixture was replaced with CHCl_3_/MeOH, the photooxidation of reactive thioanisole and 4-chlorophenyl methyl sulfide proceeded smoothly, albeit with slightly lower selectivity in the case of the halogenated sulfide (Table 1, entries 3 and 11). Diphenyl sulfide exhibited significantly slower reactivity under these conditions, with 82% conversion observed only after 16 h of irradiation (entry 25). In contrast, the oxidation of 4-aminophenyl methyl sulfide was completed within 5 h, compared to only 45% conversion when MeCN/H_2_O was used as a solvent (entry 22). These results demonstrate that both solvent mixtures are suitable for testing the oxidation of challenging substrates, thereby enhancing the flexibility of this synthetic procedure.

To further improve the sustainability of this oxidation process, we explored the efficiency of InTMPIP in aerobic photooxidation conducted in CHCl_3_/MeOH (2:1 *v*/*v*) without a photoreactor, using a commercially available 3 W domestic blue LED. As summarized in Table 1 (conditions B), significantly longer reaction times were required under these conditions (for instance, entries 4 and 5). As a result, the time required for complete conversion of sulfides became more challenging to control, and the yields of sulfoxides were slightly reduced due to the competing formation of sulfones. The oxygenation of thioanisole was successfully completed with as little as 0.0005 mol% of the PS, but it required 24 h (entry 5). The oxygenation of electron-rich 4-methoxyphenyl methyl sulfide was even slower (entry 9). This reaction produced sulfoxide and sulfone in a 10:1 ratio after 48 h of irradiation.

The oxidation of the electron-deficient 4-chloro-substituted derivative resulted in sulfoxide formation with a 97% yield after 48 h of irradiation (entry 13). Under the same conditions, the sterically hindered *ortho*-bromophenyl methyl sulfide was oxidized with only 55% conversion, selectively yielding the sulfoxide (entry 17). Increasing the PC loading tenfold accelerated both reactions, enabling the synthesis of halogenated sulfoxides within 24 h of irradiation (entries 12 and 16). Notably, these results contrasted sharply with the reactions discussed above, which were performed using dioxygen and a powerful LED, and required only 1 h under optimized conditions (entries 10 and 15).

The oxidation of diphenyl sulfide with air proved challenging. After 24 h of irradiation with 0.1 mol% of InTMPIP, only 24% of the starting compound had reacted (entry 26), and the sulfoxide-to-sulfone ratio was 93:7, despite the low conversion of sulfide. 4-Nitrophenyl methyl sulfide was oxidized using 0.05 mol% of InTMPIP (entry 21). The conversion of the starting compound was 93% after 24 h of irradiation and only the target sulfide was obtained. Surprisingly, this reaction performed using dioxygen and powerful LED afforded many byproducts, likely due to the decomposition of this nitroaromatic compounds under intense light irradiation in CHCl_3_/MeOH (entry 20).

Dialkyl sulfides exhibited greater reactivity in this aerobic oxidation compared to aryl methyl sulfides as observed in the photooxidation with dioxygen discussed above. Among these compounds, 1,4-oxathiane was the most reactive, showing quantitative conversion to sulfoxide within 1 h (TOF = 200,000 h⁻¹) (entry 31). When the PC loading was reduced tenfold, 98% of the product was obtained after 24 h (entry 32; TON = 2,000,000, TOF = 83,333 h⁻^1^; these values were calculated under the assumption of no chain reactions). Dibutyl sulfide and thian-4-one also gave 98% yield of sulfoxide after 24 h of irradiation (entries 28 and 30). Unfortunately, 2-chloroethyl ethyl sulfide, a model compound commonly used in studies on the degradation of chemical warfare agents, displayed low reactivity. It was oxidized over 48 h, producing sulfoxide and sulfone in a 91:9 ratio (entry 33).

Unexpected results were obtained when we attempted to accelerate the aerobic oxygenation of thioanisole in CHCl_3_/MeOH by conducting the reaction in an EvoluChem Photoredox Box with an 18 W, 425 nm LED (entries 6 and 7). Complete conversion of the starting compound was not achieved with either 0.0005 or 0.005 mol% of InTMPIP in 7 h. In both reactions, 38–43% of thioanisole was consumed after 2 h, but conversion only reached 43–50% after 7 h of light exposure. These results contrasted sharply with those observed in the same solvent with pure dioxygen as the oxidant (entry 3). As discussed above, this reaction was completed in just 1 h in the presence 0.005 mol% of the PC. We assume that light of increased intensity accelerates both the oxygenation reaction and the photobleaching of InTMPIP, ultimately exerting a negative effect on the progress of the aerobic photooxidation in CHCl_3_/MeOH.

Finally, we compared the performance of InTMPIP and commercially available InTPP as photosensitizers in the aerobic oxidation of thioanisole. InTPP was found to be insoluble in MeCN and MeCN/H_2_O mixtures, making these solvents inconvenient for use. Replacing InTPP with InTMPIP in the photooxidation conducted in CHCl_3_/MeOH was feasible and increased the conversion from 20% to 97% when the reaction mixture was irradiated with a 3 W LED for 24 h in the presence of 0.0005 mol% of PS. Surprisingly, when an 18 W LED was used, both PSs performed at an equal rate and both reactions were not completed in 12 h. Based on these data, the high efficiency of InTMPIP under prolonged irradiation with a 3W LED was attributed to its increased photostability in the reaction mixture.

Thus, the phosphonate-substituted imidazoporphyrin InTMPIP enables efficient and selective photooxidation of sulfides to sulfoxides using pure dioxygen in a MeCN/H_2_O (4:1 *v*/*v*) solvent mixture. This reaction can also be performed using CHCl_3_/MeOH (1:2 *v*/*v*) as a solvent. While the use of toxic chloroform is not recommended for the industrial production of fine chemicals, this option provides additional flexibility for the development of oxygenation protocols for challenging sulfides. The intensity of light is an important reaction parameter in optimizing the photooxidation of sulfides, particularly those with light-sensitive groups, where reducing light power could have a positive effect. The optimal loading of PC also depends on the light intensity employed to perform the photooxidation. Air could also serve as the oxidant for sulfides, and these reactions can be performed without specialized equipment using a 3 W domestic blue LED. However, longer irradiation times are required and decrease in selectivity could observe, which significantly reduces the practical applicability of these conditions in most of the reactions investigated in this work.

### 2.4. Photostability Studies

Comparative studies on the photostability of InTMPIP and H**_2_**TMPIP under the irradiation conditions used for photocatalytic experiments (425 nm LED, 18 W) in a MeCN/H_2_O (4:1 *v*/*v*) mixture yielded unexpected results. Both compounds exhibited relatively rapid photobleaching according to UV-vis studies, although InTMPIP was significantly more stable (a decrease of the band intensity of ~50% within 2.5 h) than H**_2_**TMPIP, which completely decomposed after just 1 h of irradiation. Replacing MeCN/H_2_O (4:1 *v*/*v*) with CHCl_3_/MeOH (1:2 *v*/*v*) accelerated photobleaching, likely due to the low photostability of chloroform (Figures S3 and S4).

When comparing the phosphonated complexes InTMPIP and InTPPP (Figures S3 and S5), we observed that the introduction of the fused imidazole linker slightly accelerated photodegradation in both solvent mixtures.

Thus, InTMPIP demonstrated high efficiency as a PC in the photooxidation of sulfides by dioxygen despite its relatively low photostability. This efficiency is likely due to its ability to effectively generate singlet oxygen, which rapidly reacts with sulfides under the investigated conditions. The rapid consumption of singlet oxygen not only produces the desired products but also helps reduce the photobleaching of the PC.

### 2.5. Grafting of InTMPIP on Mesoporous Titania

Covalent grafting of dyes onto porous titania films has been extensively studied for solar energy harvesting in dye-sensitized solar cells, as these films exhibit greater stability compared to those prepared using carboxylate anchoring groups [66]. In photocatalysis, 3D hybrid materials based on porous metal oxides [67,68] are of significant interest because they enable easy control of catalyst loading and allow for reactions and product purification to be conducted within the same apparatus as in homogeneous reactions. Recently, we developed heterogeneous catalysts by grafting homogeneous complexes with phosphonate anchoring groups onto the surface of hydrated amorphous titanium dioxide (TiO_2_) [22,40]. This solid support is cost-effective, prepared without surfactants, exhibits a high surface area (*S*_BET_ = 650–705 m^2^ g⁻^1^), and can be easily functionalized with phosphonates using a sol–gel procedure in organic solvents [69,70].

**Scheme 1:**
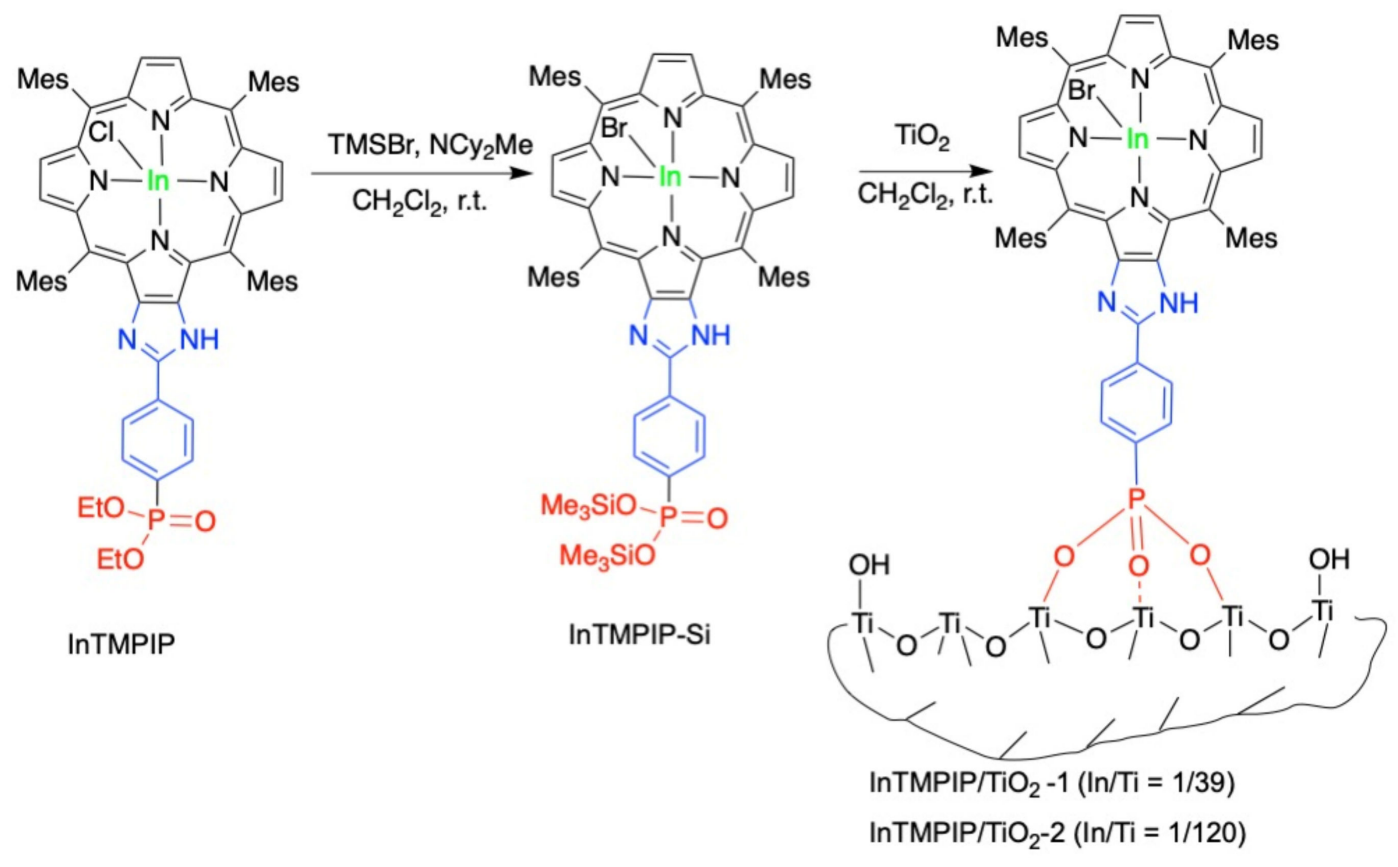
Preparation of InTMPIP/TiO_2_-1 and InTMPIP/TiO_2_-2 materials (r.t.—room temperature).

To prepare the target material, dialkyl phosphoester InTMPIP was first transformed into the reactive bis(trimethylsilyl) phosphonate InTMPIP-Si. This moisture-sensitive compound, without being isolated in pure form, was then grafted onto TiO_2_ in CH_2_Cl_2_ (Scheme 1).

This synthetic procedure, which was previously successfully used for immobilizing MnTMPIP [22], required modifications. The InTMPIP-Si, prepared by treating InTMPIP with TMSBr in CH_2_Cl_2_, was contaminated by the corresponding free-base porphyrin H_2_TMPIP-Si, likely due to the decomposition of the complex caused by traces of HBr in the reaction mixture. This side reaction was suppressed when the silylation reaction was conducted in the presence of dicyclohexylmethylamine. In line with previously reported data for Ga(III) and In(III) complexes of porphyrins [71,72], axial ligand exchange was observed during the silylation, and the corresponding bromo ligated complex was obtained in a quantitative yield, as confirmed by MALDI-TOF analysis of InTMPIP-Si.

**Figure 3:**
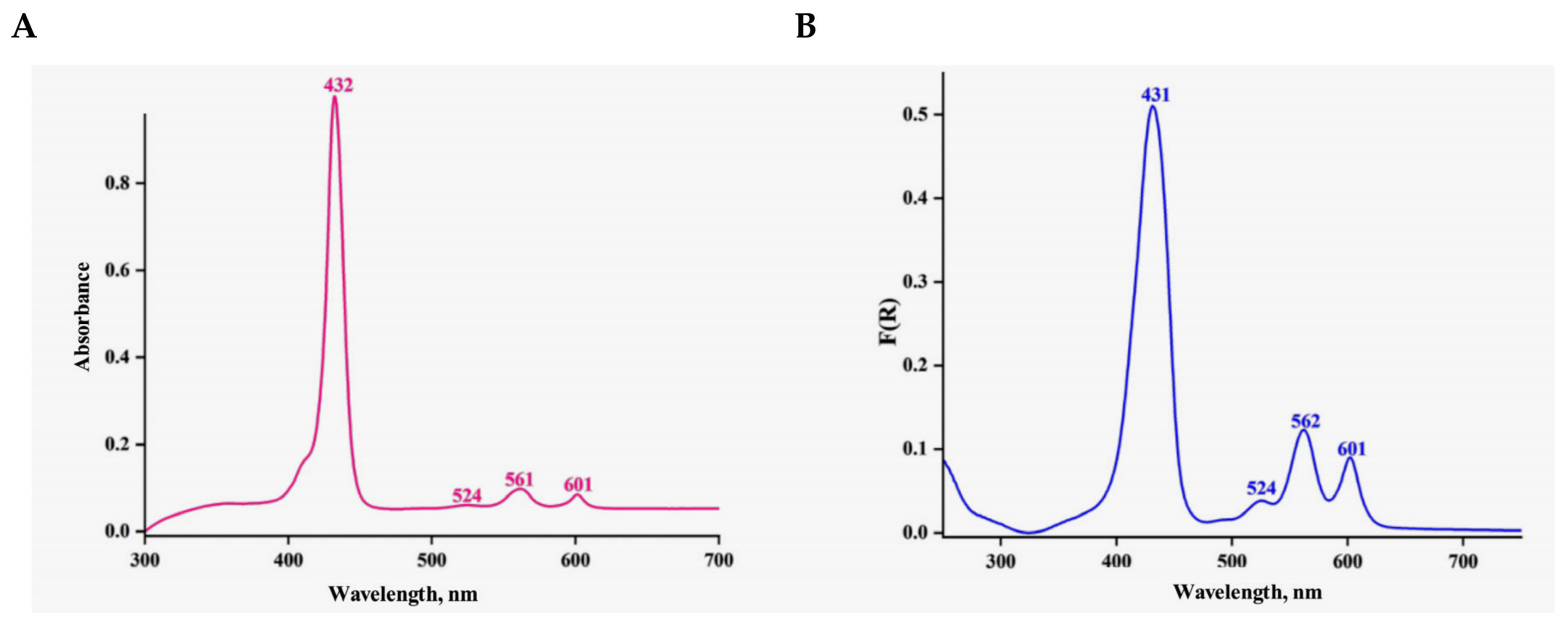
UV-vis spectrum of InTMPIP in chloroform (**A**), and diffusion reflectance spectra of InTMPIP/TiO_2_-1 (**B**).

Two materials, InTMPIP/TiO_2_-1 and InTMPIP/TiO_2_-2, were then prepared from InTMPIP-Si as shown in Scheme 1, by varying the In/Ti molar ratio in the reaction mixture from 1:30 to 1:100. Powder X-ray diffraction analysis confirmed that both materials are amorphous solids. The empirical formulas of the resulting solids were derived from the content of six elements (C, H, In, N, P, and Ti), determined by elemental analysis and ICP technique (Table S2). The In/Ti ratios in both materials were slightly lower (1:39 and 1:120, respectively) than the theoretical values, despite UV-vis analysis of the filtrates from both grafting reactions showing no detectable porphyrin derivatives. This discrepancy is not fully understood (likely because InTMPIP was obtained as a solvate), but we can confidently conclude that at least 75% of InTMPIP was grafted in each of these reactions. The intact structure of the heterogenized complex was confirmed by UV-vis diffuse reflectance and FTIR spectroscopies. The electronic absorption spectra of InTMPIP before and after immobilization were remarkably similar (Figure 3), confirming that the metalloporphyrin residue retains its structure after grafting.

In the FTIR spectra, vibration bands assigned to the porphyrin residue (720–850, 1000–1200, 1350–1610 cm^−1^) were significantly weaker compared to the Ti–OH and O–H stretching modes (3100–3700 cm^−1^) (Figure S6). A rather strong band at 1005 cm^−1^ was attributed to Ti–O–P stretching vibrations. The bonding of the phosphonate anchor to titania was also confirmed by the absence of characteristic vibration bands of the diethoxyphosphoryl group (1225–1260 cm^−1^, P=O stretch) and the phosphonic acid groups (910–920 cm^−1^, P–O–H stretch). This conclusion was further supported by ^31^P MAS NMR analysis of InTMPIP/TiO_2_-1 (Figure S7). Three broad signals, typical of titania-based non-ordered solids, were observed at 9.6, 11.4, and 18.8 ppm, with relative intensities of 2%, 92%, and 6%, respectively. Comparing these spectral data with those reported for phenylphosphonic acid grafted onto the anatase surface (PhP/An) [70], the major signal was attributed to the phosphonate group, in which each of the three oxygen atoms is bonded to titania atoms (Scheme 1). The other two signals were assigned to phosphonate groups in which one or two oxygen atoms are not involved in bonding to the titania support [70].

**Figure 4:**
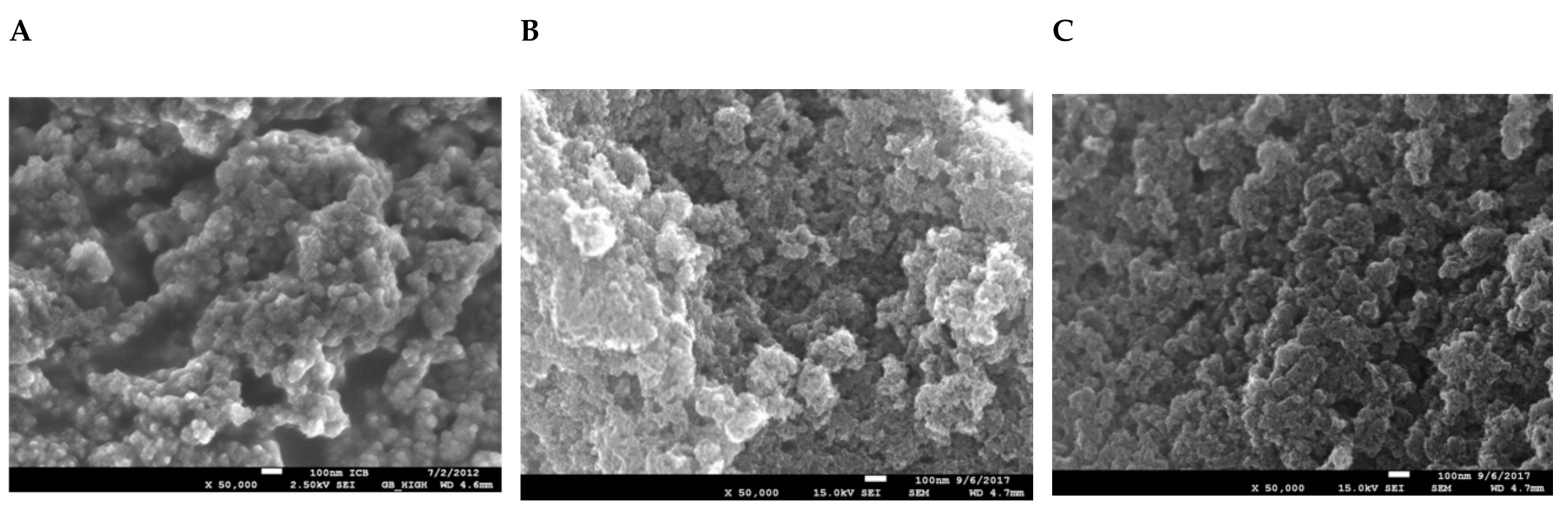
SEM images of (**A**) bare hydrated TiO_2_, (**B**) InTMPIP/TiO_2_-1, and (**C**) InTMPIP/TiO_2_-2 (the white bare corresponds to 100 nm).

The morphology of InTMPIP/TiO_2_-1 and InTMPIP/TiO_2_-2 was studied by SEM and compared to that of bare TiO_2_ (Figure 4). The bare TiO_2_ powder consists of strongly aggregated spherical nanoparticles with a relatively narrow size distribution. Grafting of InTMPIP does not alter this morphology, regardless of its quantity on the surface. Nanospheres with diameters ranging from 5 to 20 nm are irregularly aggregated and separated by large worm-like channels, with diameters on the order of hundreds of nanometers. This morphology is well-suited for catalytic applications, allowing for high catalyst loading and easy access of reagents to the catalytic centers.

N_2_ adsorption–desorption isotherms of InTMPIP/TiO_2_-1 and InTMPIP/TiO_2_-2 were recorded at 77 K (Table S3). Upon functionalization of TiO_2_ (*S*_BET_ = 705 m^2^ g^–1^, *V*_por_ = 1.25 cm^3^ g^–1^), there was no change in the shape of the isotherm; however, a marked decrease in both the BET surface area (421 m^2^ g^–1^ and 581 m^2^ g^–1^, respectively) and pore volume (0.68 cm^3^ g^–1^ and 1.10 cm^3^ g^–1^, respectively) was observed. Notably, the material with higher InTMPIP loading exhibited smaller values for both parameters. These data suggest that InTMPIP is located not only on the external surface of titania but also within its mesopores.

Both materials exhibited remarkable stability when suspended and stored in CHCl_3_ or MeOH for one month. This was confirmed by UV-vis analysis of the filtrates obtained after separation of the solids, which revealed the absence of any porphyrin derivatives in the studied solutions.

### 2.6. Photocatalytic Properties of InTMPIP/TiO_2_-2

Photocatalytic properties of InTMPIP/TiO_2_-2 were evaluated in the aerobic photooxidation of thioanisole in MeOH under irradiation with a 3 W blue LED. Using 0.013 mol% of the heterogenized catalyst, complete conversion was achieved after 12 h of irradiation, yielding the target sulfoxide in 96%. When the catalyst amount was increased to 0.09 mol% the reaction was complete in 5 h.

UV-vis spectrophotometric analysis of the reaction mixture, obtained after filtration of the heterogeneous catalyst, indicated the presence of porphyrin derivatives in the solution. The spectrum revealed characteristic Soret (432 nm) and Q bands (510–650 nm), consistent with those of InTMPIP, with the exception of several additional bands observed in the Q region (Figure S8). This suggests leaching of the grafted complex during irradiation, accompanied by partial photodegradation of the imidazoporphyrin. Notably, photodegradation products were detected exclusively in the solution, while the diffuse reflectance spectra of the recycled solid catalyst remained similar to those of InTMPIP/TiO_2_-2 (Figure S9).

The approximate amount of PS in solution was estimated based on the volume of the filtrate and the molar absorption coefficient of the Soret band observed in the spectrum of InTMPIP. According to these calculations, approximately 1 mol% of the grafted complex dissolved into the solution during the oxidation reaction. Therefore, thioanisole oxygenation could involve both homogeneous and heterogeneous PC.

**Figure 5:**
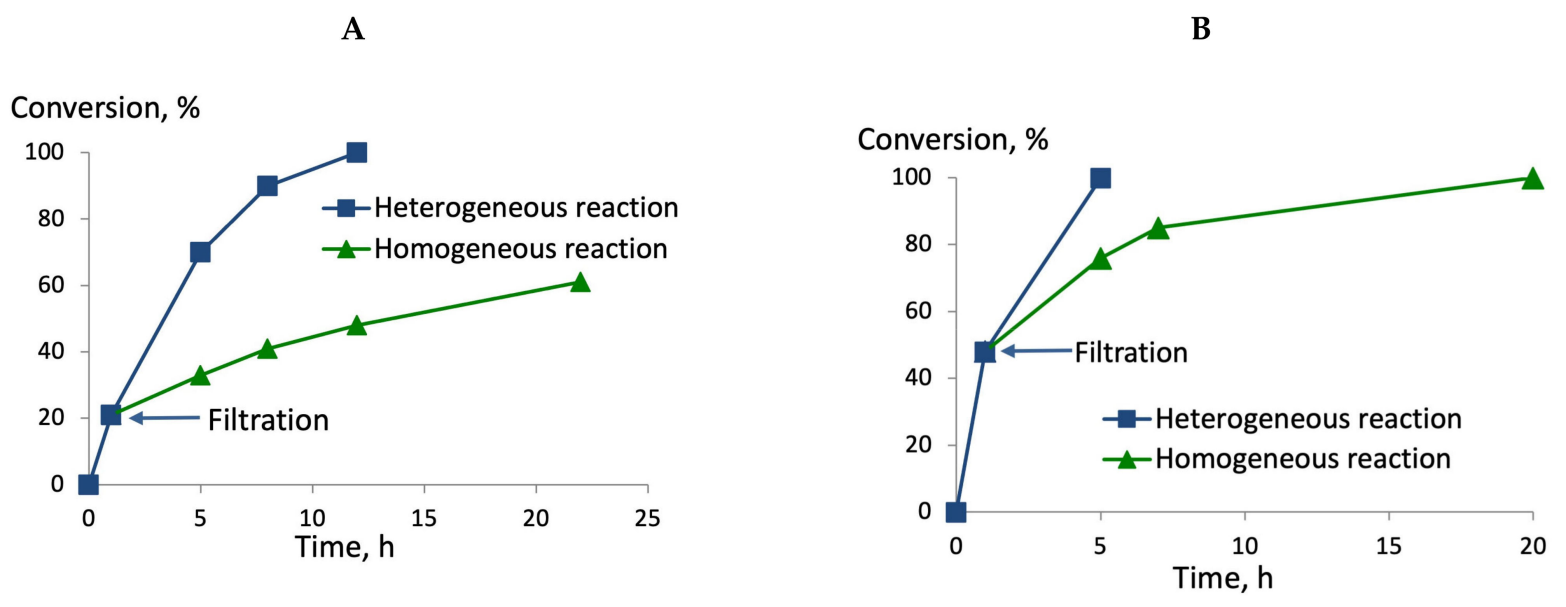
“Hot-test” experiments for the aerobic photooxidation of thioanisole in MeOH in the presence of 0.013 mol% (**A**) and 0.13 mol% (**B**) of InTMPIP/TiO_2_-2 (3 W blue LED).

To evaluate the contribution of homogeneous and heterogeneous processes in the studied photooxidation, “hot-filtration” tests were performed. After 1 h of irradiation, half of the reaction mixture was withdrawn using a syringe fitted with a 10 µm membrane filter and transferred to an identical glass vial. Then, the homogeneous and heterogeneous reactions were continued in parallel, with both vials irradiated for an additional 20–24 h and the reaction mixtures regularly analyzed by ^1^H NMR spectroscopy. Two experiments were performed, varying PS loading from 0.013 mol% to 0.13 mol%. The results, presented in Figure 5, indicate that photooxidation indeed occurs under heterogeneous and homogeneous conditions and the contribution of each pathway depends on the PS loading. At 0.013 mol% PS loading, the heterogeneous process predominates, whereas at 0.13 mol%, the homogeneous reaction becomes the major process.

In the next series of experiments, this reaction was performed in ethanol, CHCl_3_, CH_2_Cl_2_, MeCN, toluene, and THF, analyzing the filtered solutions for the presence of In(III) porphyrins. As shown in Table S4, the oxygenation proceeded much more slowly when alcohols were replaced with non-protic solvents. Unfortunately, leaching of the PS was observed in all reactions. When the light energy was reduced by replacing the blue LED with its red analogue (3 W), leaching decreased significantly but was still present, while the reaction time increased substantially (Figure S10).

Finally, we demonstrated that the washed-off In(III) complex could be grafted onto bare TiO_2_. After the reaction was completed, InTMPIP/TiO_2_-2 was filtered off for reuse in the next photocatalytic cycle. To recover the In(III) complex from the filtrate, TiO_2_ powder (100 equivalents relative to the In(III) complex introduced in the reaction) was added and the suspension was stirred for 24 h at room temperature. The precipitate, recovered InTMPIP/TiO_2_-2, was filtered, washed, and dried. UV-vis studies of the residual solution showed that the In(III) complex was grafted quantitatively. The diffuse reflectance spectrum of recovered InTMPIP/TiO_2_-2 in visible region was identical to that of InTMPIP/TiO_2_-2.

We also performed recycling experiments for the aerobic photooxidation of thioanisole in MeOH under irradiation with a 3 W blue LED for 5 h and using 0.13 mol% of heterogeneous PC. Six consecutive experiments were conducted without any loss in selectivity (Figure S11). Notably, this heterogeneous process enabled the oxygenation to be carried out in pure MeOH, eliminating the need for toxic chloroform.

## 3. Materials and Methods

### 3.1. Materials

Unless specified otherwise, all reagents and starting materials were sourced from Acros (via Thermo Fisher Scientific, Illkirch, France) or Aldrich-Sigma Co. (via Merck Co., Darmstadt, Germany) and utilized without additional purification. (5,10,15,20-Tetramesitylporphyrinato)copper(II) was supplied by PorphyChem (Dijon, France). 2-[4-(diethoxyphosphoryl)phenyl]-5,10,15,20-tetramesityl-1*H*-imidazo[4,5-*b*]porphyrin (H_2_TMPIP) was prepared on a hundred-milligram scale using a procedure previously reported by us [21]. Mesoporous hydrated titanium(IV) oxide was synthesized using a surfactant-free sol–gel method previously reported by us [40]. The titanium content in the final material was quantified using ICP-OES analysis. Dichloromethane (CH_2_Cl_2_) was purified through distillation over calcium hydride (CaH_2_).

### 3.2. Methods

Analytical thin-layer chromatography (TLC) was carried out using Merck silica gel 60 F-254 plates (precoated sheets, 0.2 mm thick, with fluorescence indicator F254; Darmstadt, Germany). The spots were visualized directly or through illumination with UV lamp (*λ* = 254 nm).

Preparative column chromatography was conducted using silica gel 60 (40–63 µm) from Merck Co. (Darmstadt, Germany).

UV-vis absorption spectra were recorded using an Agilent Cary 60 spectrophotometer (Agilent, Massy, France) and a Jasco V-550 (Jacso, Lisses, France) spectrophotometers with Suprasil 300 cuvettes (Hellma quartz cell, path length = 1 cm). Diffuse reflectance spectra of the materials were measured with a Cary 5000 UV-vis-NIR Agilent, Massy, France) spectrophotometer equipped with a Praying Mantis™ accessory (Harrick, via Agilent, Massy, France) and a Jasco V-550 (Jacso, Lisses, France) spectrophotometers. The baseline was established using a Spectralon^®^ pellet, and the corrected reflectance data (R) were converted to *F*(R) values employing the Kubelka–Munk function: F(R) = (1 − *R*^2^)/2*R*.

Fluorescence and excitation spectra were measured using a PerkinElmer LS 55 Luminescence Spectrometer (Waltham, MA, USA). Hellma fluorescence quartz cells (path length = 1 cm) were used.

FTIR spectra were acquired using either a Nicolet iS 5 spectrophotometer (Thermo Fisher Scientific, Illkirch, France) or a Bruker Vector 22 spectrophotometer (Bruker, Champs-sur-Marne, France). Solid polycrystalline samples were analyzed using a micro-ATR accessory (Pike, Madison, WI, USA).

MALDI-TOF mass spectra were recorded with a Bruker Ultraflex II LRF 2000 mass spectrometer (Bruker, Champs-sur-Marne, France) in positive ion mode, utilizing dithranol as the matrix. High-resolution mass spectrometry (HRMS-ESI) measurements were conducted using a Thermo Scientific Orbitrap Elite high-field hybrid mass spectrometer (Thermo Fisher Scientific, Illkirch, France). The samples were prepared in a methanol/chloroform (1:1, *v*/*v*) solvent mixture and analyzed in positive mode.

^1^H, ^31^P, and ^13^C NMR spectra were acquired on a Bruker Avance III 300 MHz spectrometer (Bruker, Champs-sur-Marne, France) at room temperature (at 300 MHz, 121 MHz, and 75 MHz, respectively). Chemical shifts are given in parts per million (ppm), referenced on the *δ* scale by using residual non-deuterated solvent signals as internal standard for ^1^H and ^13^C NMR spectra and external phosphonic acid (H_3_PO_4_) for ^31^P NMR spectroscopy. The coupling constants are expressed in units of frequency (Hz). In the proton numbering used in the spectra description, the atoms of the phenyl residue connected to the *meso*- position of the macrocycle are labeled as the first.

Microanalyses (CHN) were performed on a Thermo Electron Flash EA 1112 analyzer (Thermo Scientific, Lyon, France). Mn, P, and Ti contents were measured by inductively coupled plasma optical emission spectrometry (ICP-OES DUO) on an ICAP 7400 instrument from Thermo Scientific (Lyon, France). The samples were mineralized using standard procedures.

Field emission scanning electron microscopy (FESEM) was realized using a JEOL JSM 7600F instrument (JEOL (Europe) SAS, Croissy-sur-Seine, France) located in the ARCEN analysis center of the University of Bourgogne (Dijon). Images were made using GentleBeam-High SEM mode.

Nitrogen adsorption–desorption isotherms were measured with a Micromeritics ASAP 2010 analyzer (Micromeritics France SA, Mérignac, France) at 77 K with samples outgassed at 393 K under reduced pressure (105 torr) for at least 6 h. Specific surface areas were calculated by the BET method.

Powder X-ray diffraction experiments were performed with an Empyrean diffractometer (Malvern Panalytical, Vénissieux, France) in the range 3° < 2θ < 50°, using a copper anticathode X-ray tube (Cu *K*_α1_ = 1.54060 Å and Cu *K*_α__2_ = 1.54443 Å) and a X’Celerator detector outfitted with an anti-scattering slit of 5 mm. The uncrushed samples were placed between two Mylar sheets and the analysis was carried out in transmission mode using a focusing X-ray mirror equipped with fixed divergent and anti-scattering slits (aperture 0.5°) and 0.02 rad Soller slits.

All elemental analyses were performed at the “Pôle Chimie Moléculaire”, the technological platform for chemical analysis and molecular synthesis (http://www.wpcm.fr (accessed on 15 January 2025)) of the Institut de Chimie Moléculaire de l’Université de Bourgogne and Welience^TM^, a private subsidiary of the Université de Bourgogne.

### 3.3. Synthesis of {2-[4-(Diethoxyphosphoryl)phenyl]-5,10,15,20-tetramesityl-1H-imidazo[4,5-b]porphyrinato(2−)}(chloro)indium(III)

A solution of H_2_TMPIP (40 mg, 0.039 mmol), indium(III) chloride (26 mg, 0.26 mmol, 3 equiv.), and sodium acetate (95 mg, 2.59 mmol) in glacial acetic acid (18 mL) was refluxed for 3.5 h. The volatiles were removed under reduced pressure and the solid residue was purified by column chromatography using a mixture CH_2_Cl_2_/MeOH (1.5%) as an eluent. Complex InTMPIP was obtained as a dark purple solid. Yield: 95% (43.6 mg).

^1^H NMR (300 MHz, CDCl_3_, 298 K): *δ*_H_ 1.36 (t, *J* = 7.0 Hz, 6 H, Me), 1.69 (s, 3 H, 2-Me), 1.72 (s, 6 H, 2-Me), 1.73 (s, 3 H, 2-Me), 1.93 (s, 3 H, 2-Me), 1.96 (s, 3 H, 2-Me), 1.97 (s, 3 H, 2-Me), 2.01 (s, 3 H, 2-Me), 2.63 (s, 6 H, 4-Me), 2.68 (s, 3 H, 4-Me), 2.76 (s, 3 H, 4-Me), 4.07–4.24 (m,4 H, OCH_2_), 7.26 (br s, 2 H, 3-H_Ph_), 7.32 (br s, 3 H, 3-H_Ph_), 7.36 (s, 1 H, 3-H_Ph_), 7.47 (s, 1 H, 3-H_Ph_), 7.54 (s,1 H, 3-H_Ph_), 7.85 (dd, *J*_H-H_ = 8.4 Hz, ^4^*J*_P-H_ = 3.9 Hz, 2 H, 2-H_Ph(P)_), 7.95 (dd, *J*_H-H_ = 8.4 Hz, *J*_H-P_ = 12.8 Hz, 2H, 3-H_Ph(P)_), 8.62 (br s,1 H, NH), 8.84 (s, 2H, *β*-H), 8.88 and 8.93 (AB, *J* = 4.7 Hz, 2H, *β*-H), 8.94 and 8.99 (AB, *J* = 4.7 Hz, 2H, *β*-H) ppm.^31^P{H} NMR (121 MHz, CDCl_3_; 298 K): *δ*_P_ 17.96 ppm. ^13^C{H} NMR (75 MHz, CDCl_3_; 298 K): *δ*_C_ 16.4 (d, ^3^*J*_C–P_ = 6.3 Hz, 2C, Me^Et^), 21.19, 21.51(3C), 21.55 (3C), 21.64, 21.66, 21.84, 21.94, 21.96, 62.7 (d, ^2^*J*_C–P_ = 5.7 Hz, 2C, OCH_2_), 113.73, 116.98, 120.61, 120.80, 125.56, 125.76, 127.66, 127.79, 127.96, 128.14, 128.73, 129.32, 129.89, 130.45, 131.30, 131.41, 132.16, 132.48, 132.54, 132.69, 134.43, 134.47, 137.08, 137.67, 137.95, 138.00, 138.06, 138.59, 138.63, 138.76, 139.07, 139.83, 139.98, 140.44, 147.77, 148.03, 148.40, 148.48, 148.74 ppm. The observed peaks are listed, and signal intensities are provided only for the aliphatic region. UV-vis (CHCl_3_) λ_max_ [log *ε*(M^–1^ cm^–1^)]: 311 (4.40), 431 (5.55), 524 (3.67), 560 (4.29), 600 (4.04) nm. FT-IR (neat): n_max_ 3425 (s, NH), 2920 (w), 2853 (w), 1727 (s), 1609 (w), 1559 (s), 1542 (s), 1517(s), 1456 (w), 1441 (w), 1410 (s), 1378 (s), 1329 (s), 1242 (w, P=O), 1229 (w, P=O), 1194 (w),1162 (s), 1130 (w), 1051 (w), 1018 (w), 1005 (m), 966 (w), 852 (w), 831 (w), 800 (w), 750 (m), 719 (w), 665 (w).cm^−1^. HRMS (ESI+): *m*/*z* calcd. for C_67_H_65_InN_6_O_3_P ([M-Cl]^+^): 1147.38891; found: 1147.39341.

### 3.4. Determination of Emission Quantum Yields and Singlet Oxygen Quantum Yields

Fluorescence quantum yields of the compounds were measured relative to the fluorescence of H_2_TPP in toluene (*Φ*_f_ = 0.11) [73]. Sample concentrations were chosen to obtain an absorbance of 0.03–0.07, at least three measurements were performed for each sample according to the standard procedure [74]. Fluorescence quantum yields were calculated using the following formula:

$$\Phi_{s}=\Phi_{ref}\times \frac{Abs_{ref}}{Abs_{s}}\times \frac{Area_{s}}{Area_{ref}}\times {\left(\frac{\eta_{s}}{\eta_{ref}}\right)}^{2}$$

where $\Phi_{s}$ is the fluorescence quantum yield of the sample and $\Phi_{ref}$ is the fluorescence quantum yield of the standard, Abs is the absorbance at the excitation wavelength, Area is the area under the fluorescence spectrum, and η is the refractive index of the solvent.

Singlet oxygen generation quantum yields measurements were performed according to the literature [75]. To a solution of the ^1^O_2_ trap, 1,9-dimethylanthracene (DMA), with an optical density of around 1.4 in air-saturated toluene, InTMPIP was added in a quartz cuvette, and its absorbance was adjusted to around 0.1 at the wavelength of irradiation. The solution in the cuvette was irradiated with 514 nm laser (Melles Griot 43 series i laser, 543R-AP-AO1) at a constant power density of 10 mW cm^–2^. The absorption spectra of the solutions were measured every 30 s. The slope of plots of absorbance of DMA at 376 nm *vs* irradiation time for each photosensitizer was calculated.

Singlet oxygen quantum yields were calculated based on the following equation:

ΦΔ=ΦΔref  × kkref × IabsrefIabs

where *Φ*_Δ_ is the singlet oxygen quantum yield; the superscript ref stands for H_2_TPP (0.62 in toluene) [44]; *k* is the slope of the curves of DMA absorption (380 nm) change *vs* irradiation time; *I*_abs_ represents the absorption correction factor which is given by *I* = 1 − 10^–OD^ (OD is the optical density at 514 nm).

### 3.5. Photocatalytic Oxidation of Sulfides

Standard 0.01 M solutions of InTMPIP in MeCN and CHCl_3_ were prepared dissolving 0.05 mmol porphyrins in a 5 mL volumetric flask.

*General procedure of sulfoxidation by dioxygen in MeCN/H_2_O or CHCl_3_/MeOH (conditions A).* A glass vial equipped with a magnetic stir bar was fulfilled 3 times with dioxygen using vacuum–O_2_ cycles. This vial was charged with 0.5 mmol of sulfide (see Table 1) and calculated amount (see Table 1) of standard solution of the photocatalyst. Then, solvents were added to obtain solution of sulfide and InTMPIP in 1.25 mL of the MeCN/H_2_O (4:1, *v*/*v*) or CHCl_3_/MeOH (1:2 *v*/*v*) solvent mixture. The reaction was stirred and irradiated with blue LED (425 nm, 18 W) under O_2_ (balloon, 0.75 L) in PhotoRedOx^TM^ Box (HepatoChem, Beverly, USA) photoreactor. The reaction was monitored by ^1^H NMR spectroscopy. When the reaction was complete, dichloromethane was added (10 mL) and the aqueous layer was separated. The organic phase was dried over Na_2_SO_4_ and evaporated under reduced pressure. The yield and purity of the products were determined by ^1^H NMR using biphenyl as an internal standard. The results are summarized in the Table 1.

*General procedure of aerobic sulfoxidation in CHCl_3_/MeOH (conditions B).* A glass vial equipped with a magnetic stir bar was charged with 0.5 mmol of sulfide (see Table 1) and a calculated amount (see Table 1) of standard solution of the photocatalyst. Then, CHCl_3_ and MeOH were added to obtain the reaction mixture in 1.25 mL of the CHCl_3_/MeOH (1:2 *v*/*v*) mixture. The reaction was stirred at room temperature and irradiated with a blue LED (3 W, positioned 3 cm away) under air. The yield and purity of the products were determined by GS-MS analysis using trimesitylene as an internal standard. The results are summarized in the Table 1.

Similar oxidation reactions were performed replacing 3 W LED by 18 W LED (HepatoChem, Beverly, USA) (conditions C). When the reaction was complete, the mixture was evaporated. The yield and purity of the products were determined by ^1^H NMR using biphenyl as an internal standard. The results are summarized in the Table 1.

The experiments with the heterogeneous catalyst InTMPIP/TiO_2_-2 and recycled photocatalyst were performed using the same procedure (conditions B) in methanol. The recycling reactions were scaled up to 1.5 mmol of thioanisole and conducted with 0.13 mol% of PS, with the reaction mixtures being irradiated for 5 h. Then, the heterogeneous PC was filtered and washed with CH2Cl2 (3 × 5 mL), and the recovered PC was introduced in the next catalytic cycle. The filtrate was evaporated and analyzed by ¹H NMR spectroscopy, with biphenyl added as an internal standard.

All sulfoxides obtained in this work have been previously reported, and their spectra were consistent with those found in the literature.

*Methyl phenyl sulfoxide* [64]. ^1^H NMR (300 MHz, CDCl_3_, 298 K): *δ*_H_ 2.75 (s, 3H, CH_3_), 7.51–7.59 (m, 3H, Ar), 7.66–7.70 (m, 2H, Ar) ppm.

*4-Methoxyphenyl methyl sulfoxide* [64]. ^1^H NMR (300 MHz, CDCl_3_, 298 K): *δ*_H_ 2.72 (s, 3H, CH_3_), 3.88 (s, 3H, OCH_3_), 7.03−7.08 (m, 2H, Ar), 7.59−7.68 (m, 2H, Ar) ppm.

*4-Chlorophenyl methyl sulfoxide* [64]. ^1^H NMR (300 MHz, CDCl3, 298 K): *δ*_H_ 2.74 (s, 3H, CH3), 7.51−7.56 (m, 2H, Ar), 7.59−7.66 (m, 2H, Ar) ppm.

*2-Bromophenyl methyl sulfoxide* [64]. ^1^H NMR (300 MHz, CDCl_3_, 298 K): *δ*_H_ 2.84 (s, 3H, CH_3_), 7.40 (ddd, *J* = 8.0, 7.4, 1.7 Hz, 2H, Ar), 7.57−7.63 (m, 2H, Ar), 7.95−7.99 (m, 2H, Ar) ppm.

*Methyl 4-nitrophenyl sulfoxide* [64]. ^1^H NMR (300 MHz, CDCl_3_, 298 K): *δ*_H_ 2.82 (s, 3H, CH_3_), 7.84−7.88 (m, 2H, Ar), 8.40−8.45 (m, 2H, Ar) ppm.

*4-Aminophenyl methyl sulfoxide* [64]. ^1^H NMR (300 MHz, CDCl_3_, 298 K): *δ*_H_ 2.70 (s, 3H, CH_3_), 6.76−6.80 (m, 2H, Ar), 7.45−7.50 (m, 2H, Ar) ppm.

*4-Methyl sulfinyl benzonitrile* [76]. ^1^H NMR (300 MHz, CDCl_3_, 298 K): *δ*_H_ 2.79 (s, 3H, CH_3_), 7.77−7.80 (m, 2H, Ar), 7.84−7.88 (m, 2H, Ar) ppm.

*Diphenyl sulfoxide* [76]. ^1^H NMR (300 MHz, CDCl_3_, 298 K): *δ*_H_ 7.29–7.43 (m, 6H, Ar), 7.56–7.63 (m, 4H, Ar) ppm.

*Dibutyl sulfoxide* [64]. ^1^H NMR (300 MHz, CDCl_3_, 298 K): *δ*_H_ 0.98 (t, *J* = 7.3 Hz, 6H, CH_3_), 1.41–1.61 (m, 4H, CH_2_), 1.71–1.82 (m, 4H, CH_2_), 2.60–2.76 (m, 4H, CH_2_) ppm.

*Thian-4-one S-oxide* [77]. ^1^H NMR (300 MHz, CDCl_3_, 298 K): *δ*_H_ 2.54–2.61 (m, 2H, CH_2_), 2.84–2.97 (m, 2H, CH_2_), 3.31–3.43 (m, 4H, 2CH_2_) ppm.

*1,4*-Oxathiane 4-oxide [78]. ^1^H NMR (300 MHz, CDCl_3_, 298 K): *δ*_H_ 2.65–2.67 (m, 2H, CH_2_), 2.84–2.90 (m, 2H, CH_2_), 3.72–3.79 (m, 2H, CH_2_), 4.24–4.33 (m, 2H, CH_2_) ppm.

### 3.6. Synthesis of Heterogenized Catalysts

A dry Schlenk tube was charged with dried phosphodiester InTMPIP (60 mg, 0.0507 mmol) and dry CH_2_Cl_2_ (50 mL) under argon. Then, TMSBr (100 equiv.) and dicyclohexylmethylamine (100 equiv.) were added via a syringe and the resulting mixture was stirred for 48 h at room temperature. The reaction was monitored by MALDI-TOF mass spectrometry to ensure a complete conversion has occurred before evaporation of volatiles under reduced pressure. Then, 50 mL of dry CH_2_Cl_2_ were introduced into the Schlenk tube with a syringe, followed by the addition of mesoporous hydrated TiO_2_ (5 mmol, 100 equiv.) under an argon stream. The suspension was stirred for 48 h at room temperature. Then, the solid was collected by centrifugation, thoroughly washed with THF (2 × 20 mL), water (15 mL), MeOH (3 × 15 mL), and ether (2 × 15 mL). The material InTMPIP/TiO_2_-2 thus obtained was dried for 24 h at 80 °C under reduced pressure (2 mmHg). Yield: 450 mg.

The solid InTMPIP/TiO_2_-1 with a high content of InTMPIP (ratio of In(TMPIP):TiO_2_ was 1:30) was prepared according to this procedure.

## 4. Conclusions

In this work, a phosphonate-substituted imidazo[4,5-*b*]porphyrin InTMPIP was synthesized and investigated as both a homogeneous and heterogeneous PC in the oxidation of sulfides to sulfoxides.

The insertion of In(III) ions into imidazoporphyrin H_2_TMPIP proceeded smoothly, yielding the target complex InTMPIP quantitatively. InTMPIP exhibits excellent solubility in toluene, various polar organic solvents, and even aqueous solutions, due to the presence of a bulky and hydrophilic phosphonate group at the periphery of the tetrapyrrolic macrocycle. The complex efficiently generates singlet oxygen, with a quantum yield reaching 92% in toluene. These properties enable its efficient use as a PS in oxidation reactions involving dioxygen.

In the course of this work, we identified practical conditions for the selective photooxidation of sulfides to sulfoxides using dioxygen in a “green” solvent mixture MeCN/H_2_O (4:1 *v*/*v*). Additionally, this reaction can be carried out in a CHCl_3_/MeOH solvent system, offering flexibility in optimizing photooxidation for challenging sulfides. While aerobic oxidation using this PC and a domestic blue LED (3 W) is feasible, the reaction proceeds too slowly for practical applications.

This work is part of our systematic investigations of phosphonate-substituted photocatalysts and reinforces the potential of such dyes in developing “green” oxidation methods. The photooxidation of sulfides to sulfoxides by dioxygen proceeds via different mechanisms, and the choice of optimal conditions strongly depends on the nature of the sulfide. Expanding the library of efficient PCs is thus crucial for broadening scope of this reaction, especially for the late-stage synthesis of pharmaceuticals, where sulfides with sensitive functional groups must be transformed into target compounds with high yields. Despite the challenging synthesis of imidazo[4,5-*b*]porphyrin H_2_TMPIP, the derived InTMPIP complex could be of interest for oxidizing specific sulfides. In contrast, for more reactive aryl and alkyl sulfides, readily available and soluble complexes like MTPPP (M = Pd(II), In(III)) are likely the better choice.

Our studies also highlighted the critical role of light intensity as a reaction parameter. While photooxidations are generally accelerated without a decrease in selectivity when the LED power is increased, this can have a negative impact when substrates contain light-sensitive functional groups or when using PCs with limited photostability.

We also explored the immobilization of InTMPIP on hydrated mesoporous titanium dioxide using a phosphonate anchoring group. This approach, which has proven highly effective for transition metal-catalyzed cross-coupling reactions and the selective oxidation of sulfides to sulfoxides by the dioxygen/IBA system, demonstrated limited utility for photooxidation reactions due to the relatively rapid leaching of the PS under irradiation with blue and even red light. We anticipate that this issue can be mitigated by increasing the number of phosphonate anchoring groups in porphyrin molecules. Currently, we are working on incorporating MTPPP (M = In(III), Pd(II)) into a titania matrix using a sol–gel process. These materials are expected to more closely achieve the desired properties of reusable heterogeneous porphyrin catalysts.

## Figures and Tables

**Table 1:** Photooxidation of sulfides in the presence of InTMPIP.

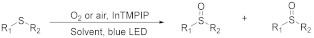								
Entry	Sulfide	Photocatalyst (mol%)	Solvent	Reaction Conditions ^1^	Time (h)	Conversion^2^ (%)	Yield ^2^ (%)	
							Sulfoxide	Sulfone
1 2 3 4 5 6 7	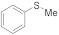	0.05 0.005 0.005 0.1 0.0005 0.005 0.0005	MeCN/H_2_O MeCN/H_2_O MeOH/CHCl_3_ MeOH/CHCl_3_ MeOH/CHCl_3_ MeOH/CHCl_3_ MeOH/CHCl_3_	A A A B B C C	1 1 1 5 24 2 7 2	100 100 100 100 97 38 50 43 45	98 98 99 92 97 100 100 100 100	2 2 1 8 3 0 0 0 0
8 9	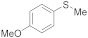	0.005 0.0005	MeCN/H_2_O MeOH/CHCl_3_	A B	1 24 48	100 66 100	98 95 91	2 5 9
10 11 12 13	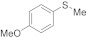	0.005 0.005 0.005 0.0005	MeCN/H_2_O MeOH/CHCl_3_ MeOH/CHCl_3_ MeOH/CHCl_3_	A A B B	1 1 24 24 48	100 100 100 57 100	99.5 97 97 96 97	0.5 3 3 4 3
14	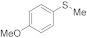	0.005	MeCN/H_2_O	A	1	100	98	2
15 16 17	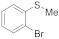	0.005 0.005 0.0005	MeCN/H_2_O MeOH/CHCl_3_ MeOH/CHCl_3_	A B B	1 24 24 48	100 100 23 55	100 100 100 100	0 0 0 0
18 19 20 21	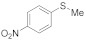	0.005 0.05 0.005 0.05	MeCN/H_2_O MeCN/H_2_O MeOH/CHCl_3_ MeOH/CHCl_3_	A A C B	20 12 20 20 24	40 55 100 68 ^3^ 93	100 100 98 37 ^4^ 100	0 0 2 0 0
22 23	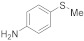	0.005 0.005	MeCN/H_2_O MeOH/CHCl_3_	A A	5 10 5	45 77 100	100 100 100	0 0 0
24 25 26	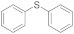	0.005 0.005 0.1	MeCN/H_2_O MeOH/CHCl_3_ MeOH/CHCl_3_	A A B	3 6 3 16 24	53 100 63 82 24	98 98 93	2 2 7
27 28	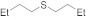	0.005 0.005	MeCN/H_2_O MeOH/CHCl_3_	A B	1 24	100 100	95 98	5 2
29 30	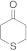	0.005 0.005	MeCN/H_2_O MeOH/CHCl_3_	A B	1 24	100 83	100 98	0 2
31 32	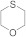	0.0005 0.00005	MeOH/CHCl_3_ MeOH/CHCl_3_	B B	1 24	100 100	100 98	0 2
33	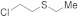	0.00005	MeOH/CHCl_3_	B	48	100	91	9

^1^ Reaction conditions: A: 0.5 mmol of sulfide and InTMPIP in a solvent mixture specified in the table (1.25 mL; MeCN/H_2_O 4:1 *v*/*v*, MeOH/CHCl_3_ 2:1 *v*/*v*) were stirred and irradiated with a 425 nm LED (18 W) at room temperature (r.t.) under a dioxygen atmosphere (0.75 L balloon) in an EvoluChem Photoredox box. B: 0.5 mmol of sulfide and InTMPIP in MeOH/CHCl_3_ (2:1 *v*/*v*; 1.25 mL) were stirred in air irradiating the reaction mixture with a blue LED (3 W) at room temperature. C: 0.5 mmol of sulfide and InTMPIP in MeOH/CHCl_3_ (2:1 *v*/*v*; 1.25 mL) were stirred in air upon irradiation with a 425 nm LED (18 W) at room temperature in an EvoluChem Photoredox box. ^2^ Conversion and selectivity (the ratio of sulfoxide to sulfone multiplied by 100%) were determined by NMR (for reactions with dioxygen) and GC-MS (for reactions in air) analyses of reaction mixtures using biphenyl and mesitylene, respectively, as internal standards.^3^ Oily compounds were also obtained but only soluble products were investigated. At least two soluble byproducts were observed in the ^1^H NMR spectra. ^4^ The yield of the sulfoxide could be significantly overestimated since oily products were not taken into account, and numerous signals with low intensity in the aromatic region were not accounted for.

## Data Availability

Data are contained within the article and Supplementary Materials.

## Supplementary Materials

The following supporting information can be downloaded at: https://www.mdpi.com/article/10.3390/molecules30040864/s1, Figure S1. Normalized UV-vis spectra of InTMPIP, InTPP, and InTPPP in MeCN/H_2_O (4:1 *v*/*v*); Table S1. Optical properties of H_2_TMPIP, InTMPIP, and H_2_TPP in toluene; Figure S2. Photosensitized oxidation of 1,9-dimethylanthracene (DMA) in the presence of InTMPIP in air saturated acetonitrile solution irradiated with 514 nm laser (10 mW cm^−2^). Inset: change in absorbance at 380 nm with time; Figure S3. UV-vis spectra of a InTMPIP solution in MeCN/H_2_O (4:1 *v*/*v*) irradiated with a 425 nm LED (18 W) in an EvoluChem Photoredox Box; Figure S4. UV-vis spectra of a InTMPIP solution in CHCl_3_/MeOH (1:2 *v*/*v*) irradiated with a 425 nm LED (18 W) in an EvoluChem Photoredox Box; Figure S5. UV-vis spectra of a InTPPP solution in MeCN/H_2_O (4:1 *v*/*v*) irradiated with a 425 nm LED (18 W) in an EvoluChem Photoredox Box; Table S2. Chemical composition of solids prepared by grafting InTMPIP; Figure S6. FTIR spectra of InTMPIP-TiO_2_-1, InTMPIP, and hydrated TiO_2_; Figure S7. ^31^P MAS NMR spectra of InTMPIP-TiO_2_-1; Table S3. BET surface area, total pore volume and pore diameter for hydrated titania and heterogenized catalysts obtained in this work; Figure S8. UV-vis studies of the filtrate obtained after completing the aerobic photooxidation of thioanisole (blue LED, 3 W) in the presence of InTMPIP/TiO_2_-2 in MeOH, followed by filtration of the PS. Color code: blue line—filtrate from the reaction; red line—InTMPIP; green line—filtrate after stirring for 24 h with a fresh portion of TiO_2_; Table S4. Aerobic photooxidation of thioanisole in the presence of InTMPIP/TiO_2_-2; Figure S9. Diffusion reflectance spectra of InTMPIP/TiO_2_-2 before and after the aerobic photooxidation of thioanisole (blue LED, 3 W) in MeOH. Inlet: UV-vis spectrum of InTMPIP in chloroform; Figure S10. “Hot-test” experiments for aerobic photooxidation of thioanisole in MeOH in the presence of 0.013 mol% (A) and 0.13 mol% (B) InTMPIP/TiO2-2 (red LED, 3 W); Figure S11. Recycling of InTMPIP/TiO2-2 in the aerobic photooxidation of thioanisole (1.5 mmol of thioanisole, 0.13 mol% of PS; MeOH (3.6 mL), blue LED 3 W, 5 h); Figure S12. Selected regions of ^1^H NMR spectrum of InTMPIP (300 MHz, CDCl_3_, 298 K); Figure S13. ^31^P NMR spectrum of InTMPIP (121 MHz, CDCl_3_, 298 K); Figure S14. ^13^C NMR spectrum of InTMPIP (75 MHz, CDCl_3_, 298 K); Figure S15. HRMS-ESI mass spectrum of InTMPIP; Figure S16–S26. ^1^H NMR spectra (300 MHz, CDCl_3_, 298 K) of the reaction mixtures obtained in the photooxidation of sulfides.
