# Supplementary material for: Indium Imidazo[4,5,-b]porphyrins as Photocatalysts for Oxidation of Sulfides
Source: Molecules. 2025 Feb 13;30(4):864. doi: 10.3390/molecules30040864 (PMC11857907; doi:10.3390/molecules30040864)
Supplement: Supplementary file 1 [file molecules-30-00864-s001.zip › molecules-3464447-supplementary.pdf]

Supporting Information

Table of contents

1. UV-vis spectroscopic studies of InTMPIP, InTPP and InTPPP 2
2. Studies of singlet oxygen generation by InTMPIP 3
3. UV-vis spectroscopic studies of photostability of InTMPIP and InTPPP 4
4. Characterization of hydrid materials 7
5. Photooxidation of thioanisole catalyzed by InTMPIP-TiO_2-2 10
6. NMR and HRMS-ESI spectra of complex InTMPIP 13
7. NMR spectra of the reaction mixtures obtained in the photooxidation of sulfides 15

1. UV-vis spectroscopic studies InTMPIP, InTPP and InTPPP

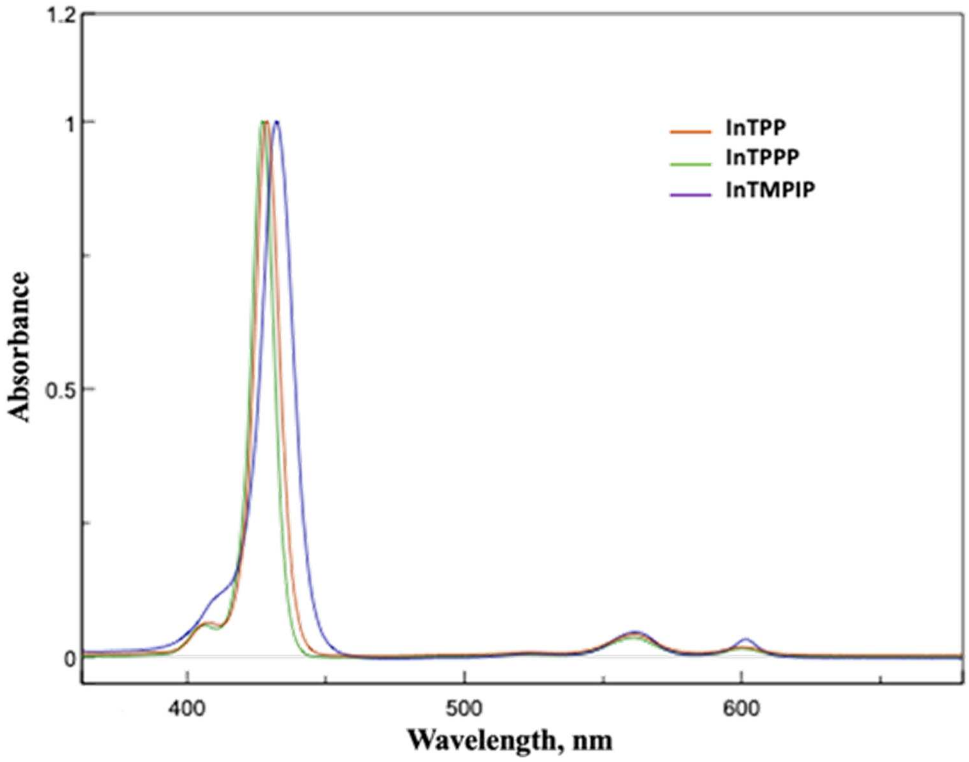

Figure S1. Normalized UV-vis spectra of InTMPIP, InTPP and InTPPP in MeCN/H_2O (4:1 v/v).

Table S1. Optical properties of H_2TMPIP, InTMPIP and H_2TPP in toluene.

Compound	λ_{abs}	λ_{em}	Φ_f	Φ_Δ
H_2TPP	419, 515, 548, 592, 649	654, 718	0.11[1]	0.62[2]
H_2TMPIP	420, 515, 548, 589, 650	652, 714	0.101	0.65
InTMPIP	432, 524, 562, 601	606, 662	0.015	0.92

[1] Taken from P. G. Seybold, M. Gouterman, *J. Mol. Spectrosc.*, **1969**, *31*, 1-10. [2] Taken from F. Wilkinson, P. Helman, A.B. Ross, *J. Phys. Chem. Ref. Data*, **1993**, *22*, 113.

2. Studies of singlet oxygen generation by InTMPIP

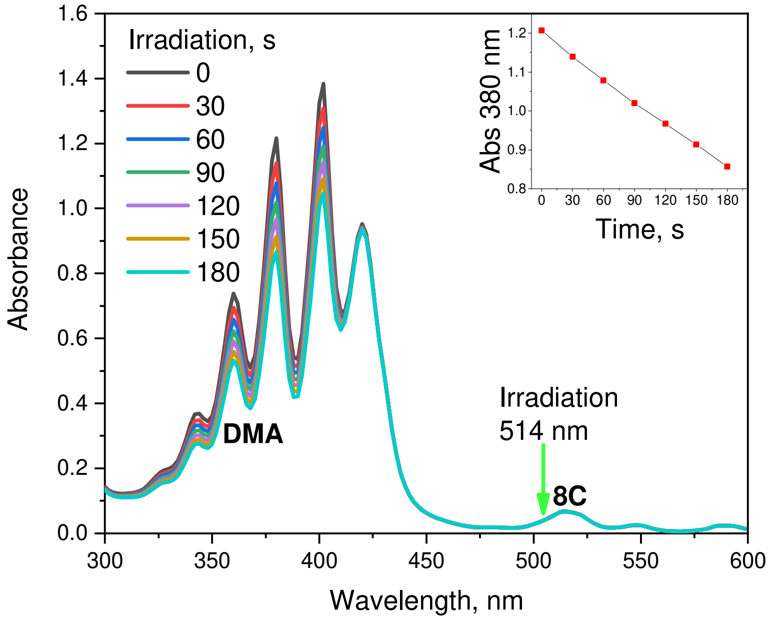

Figure S2. Photosensitized oxidation of 1,9-dimethylanthracene (DMA) in the presence of InTMPIP in air-saturated acetonitrile solution irradiated with a 514 nm laser (10 mW cm^{-2}). Inset: change of absorbance at 380 nm with time.

3. UV-vis spectroscopic studies of photostability of InTMPIP and InTPPP

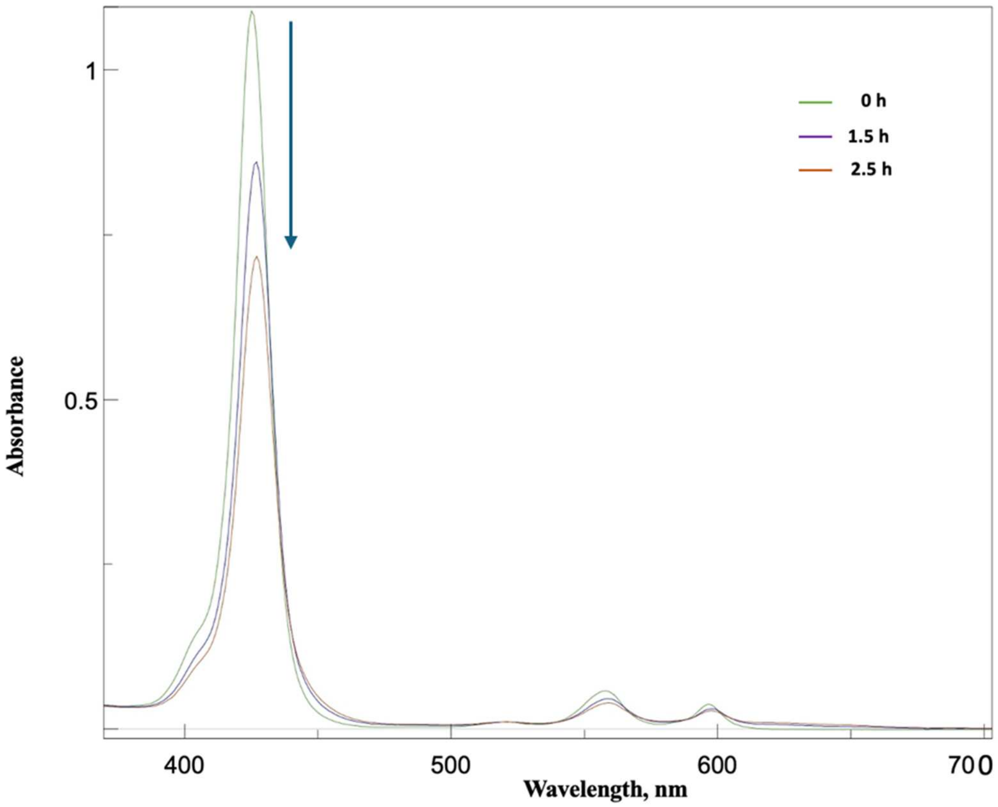

Figure S3. UV-vis spectra of an InTMPIP solution in MeCN/H_2O (4:1 v/v) irradiated with a 425 nm LED (18 W) in an EvoluChem Photoredox Box.

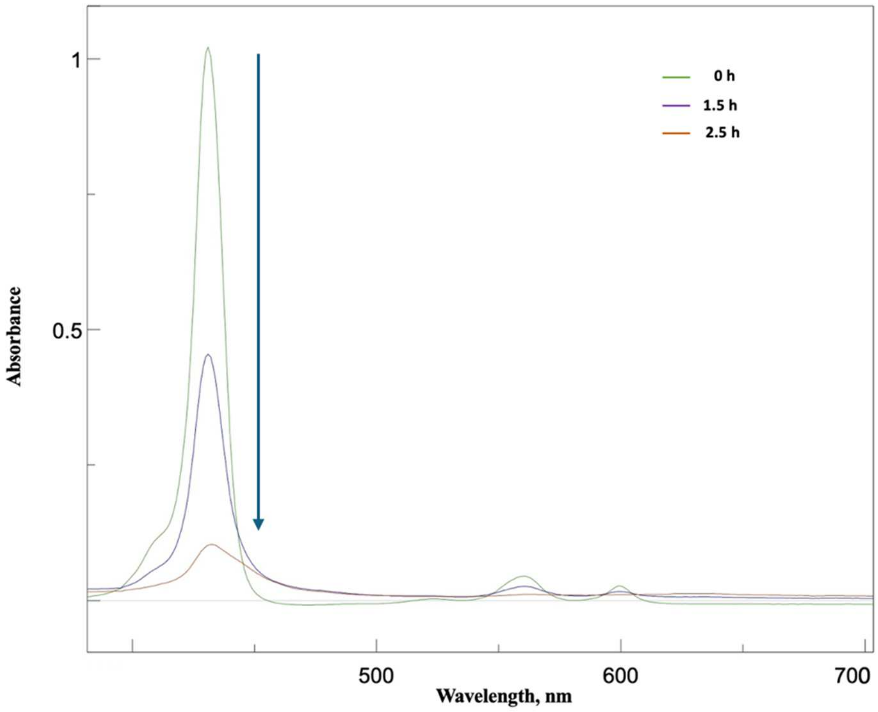

Figure S4. UV-vis spectra of an InTMPIP solution in $CHCl_3$/MeOH (1:2 v/v) irradiated with a 425 nm LED (18 W) in an EvoluChem Photoredox Box.

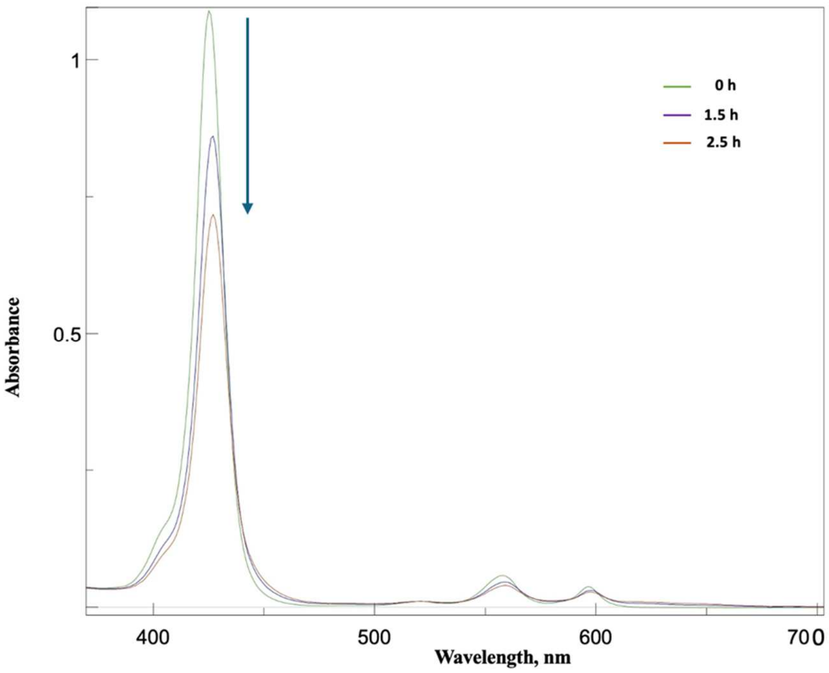

Figure S5. UV-vis spectra of an InTPPP solution in MeCN/H_2O (4:1 v/v) irradiated with a 425 nm LED (18 W) in an EvoluChem Photoredox Box.

4. Characterization of hybrid materials

Table S2. Chemical composition of solids prepared by grafting InTMPIP.[1]

Entry	Material	Calculated formula		Elemental analysis						
				C/N	N/P	Ti/P	Ti/N	Ti/In	N/In	P/In
1	**In(TMPIP)/TiO₂-1**		found	13.4	5.4	34.4	6.4	40.3	6.3	1.2
		$(C_{63}H_{55}N_6PInBrO_2)(TiO_2)_{39}(H_2O)_{43}(C_3H_7OH)_4$	calcd.	13.2	6.0	38.0	6.4	38.0	6.0	1.0
2	**In(TMPIC)/TiO₂-2**		found	20.8	4.2	117.7	27.8	128.1	4.5	1.1
		$(C_{64}H_{55}N_6PInBrO_2)(TiO_2)_{120}(H_2O)_{60}(C_3H_7OH)_{15}$	calcd.	21.2	4.0	120.0	30.0	120.0	4.0	1.0

1 Calculations were made using the data from C, N and H analyses (Thermo Electron Flash EA 1112 analyzer) and P, Ti and In analyses (ICP-OES ICAP 7400 instrument).

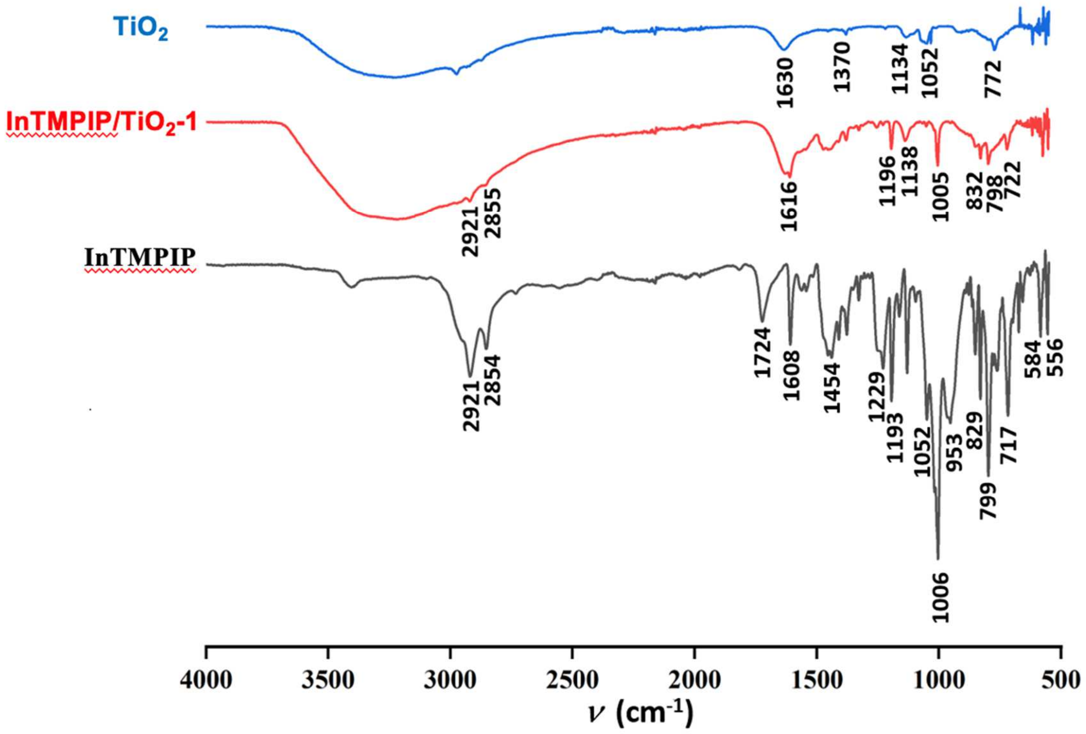

Figure S6. FTIR spectra of InTMPIP-TiO_2-1, InTMPIP and hydrated mesoporous TiO_2.

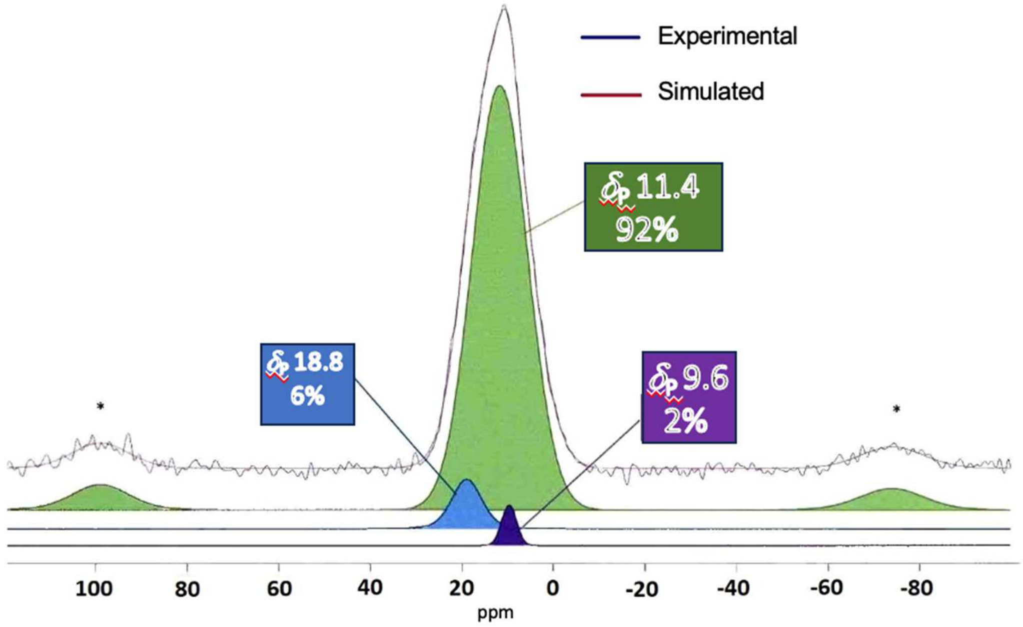

Figure S7. ^{31}P MAS NMR spectra of InTMPIP-TiO_2-1.

Table S3. BET surface area, total pore volume and pore diameter for hydrated titania and heterogenized catalysts obtained in this work.

Entry	Solid	BET surface area [m^2 g^{-1}]	Total pore volume [m^3 g^{-1}]	Pore diameter [Å]
1	**TiO_2**	705	1.25	20–150
2	**InTMPIP/TiO_2-1**	421	0.68	20–120
5	**InTMPIP/TiO_2-2**	581	1.10	20–120

5. Photooxidation of thioanisole catalyzed by InTMPIP/TiO_2-2

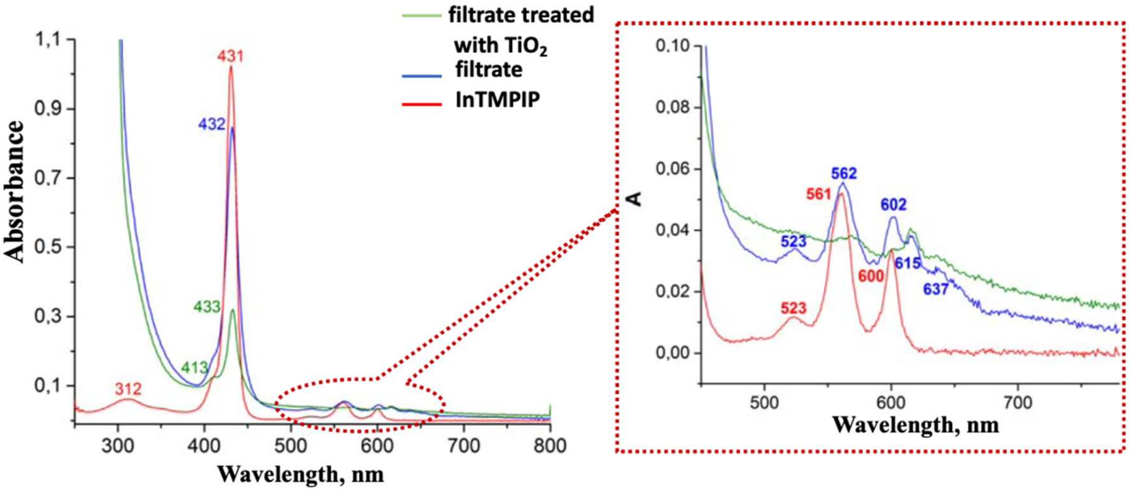

Figure S8. UV-vis studies of the filtrate obtained after completing the aerobic photooxidation of thioanisole (blue LED, 3 W) in the presence of InTMPIP/TiO_2-2 in MeOH, followed by filtration of the PS. Color code: blue line – filtrate from the reaction; red line – InTMPIP; green line – filtrate after stirring for 24 hours with a fresh portion of TiO_2.

Table S4. Aerobic photooxidation of thioanisole in the presence of InTMPIP/TiO_2-2.[1]

Ent-ry	Solvent	Temps (h)	Conversion[2] (%)	Selectivity[2] (%)	
				Sulfoxide	Sulfone
1	MeOH	5	100	96	4
2	EtOH	4	94	94	4
4	$CHCl_3$	24	100	94	6
6	CH_2Cl_2	24	90	94	6
7	MeCN	24	85	89	11
8	Toluene	24	45	91	9
9	CCl_4	24	42	86	14

[1] Reaction conditions: 0.5 mmol of thioanisole and 0.09 mol% (calculated based on the of InTMPIP) of InTMPIP/TiO_2-2 in the solvent (1.25 mL) were stirred in air while the reaction mixture was irradiated the reaction mixture with a blue LED (3 W) at room temperature. [2] Conversion and selectivity (the ratio of sulfoxide to sulfone multiplied by 100%) were determined by ^{1}H NMR spectroscopy using mesitylene as an internal standard.

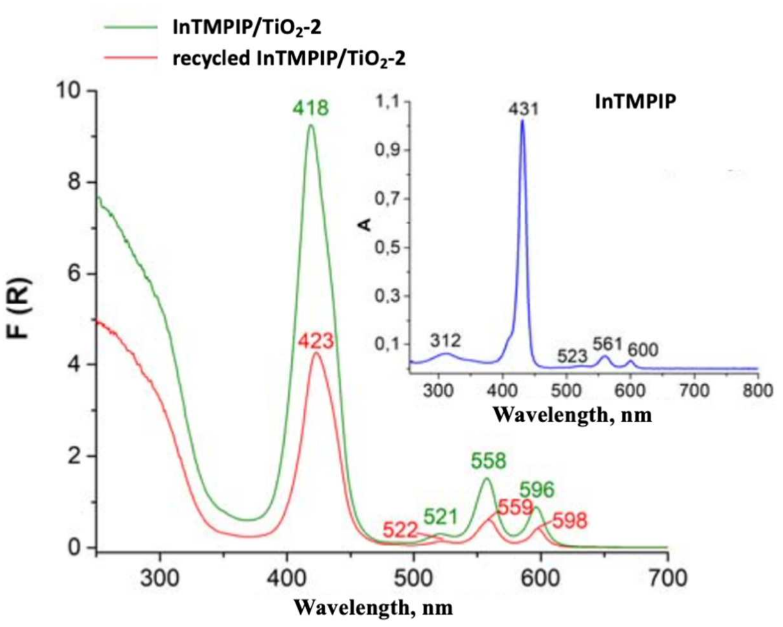

Figure S9. Diffuse reflectance spectra of InTMPIP/TiO_2-2 before and after the aerobic photooxidation of thioanisole (blue LED, 3 W) in MeOH. Inset: UV-vis spectrum of InTMPIP in chloroform.

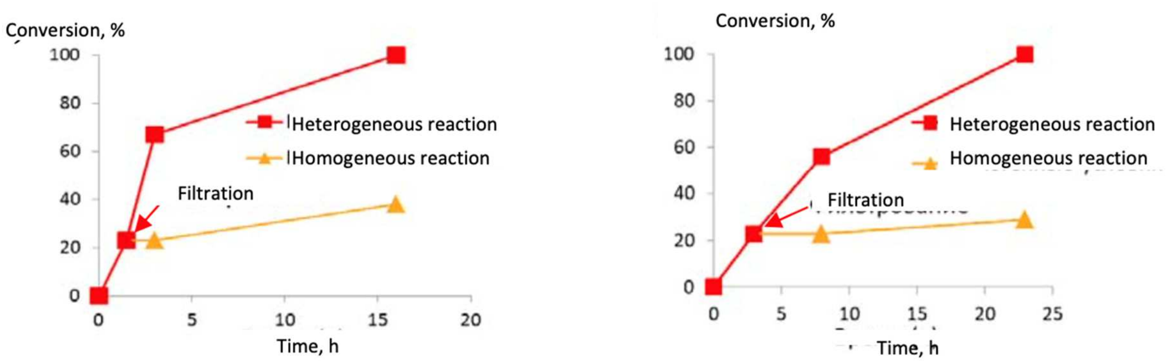

Figure S10. 'Hot-test' experiments for aerobic photooxidation of thioanisole in MeOH in the presence of 0.013 mol% (A) and 0.13 mol% (B) of InTMPIP/TiO_2-2 (red LED, 3 W).

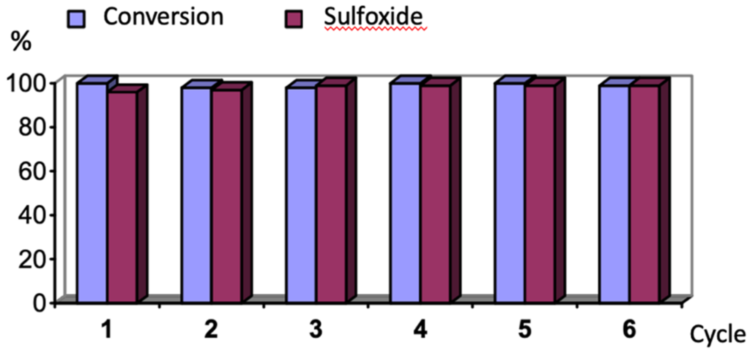

Figure S11. Recycling of InTMPIP/TiO_2-2 in the aerobic photooxidation of thioanisole (1.5 mmol of thioanisole, 0.13 mol% of PC; MeOH (3.6 mL), blue LED 3 W, 5 h).

6. NMR and HRMS-ESI spectra of complex InTMPIP

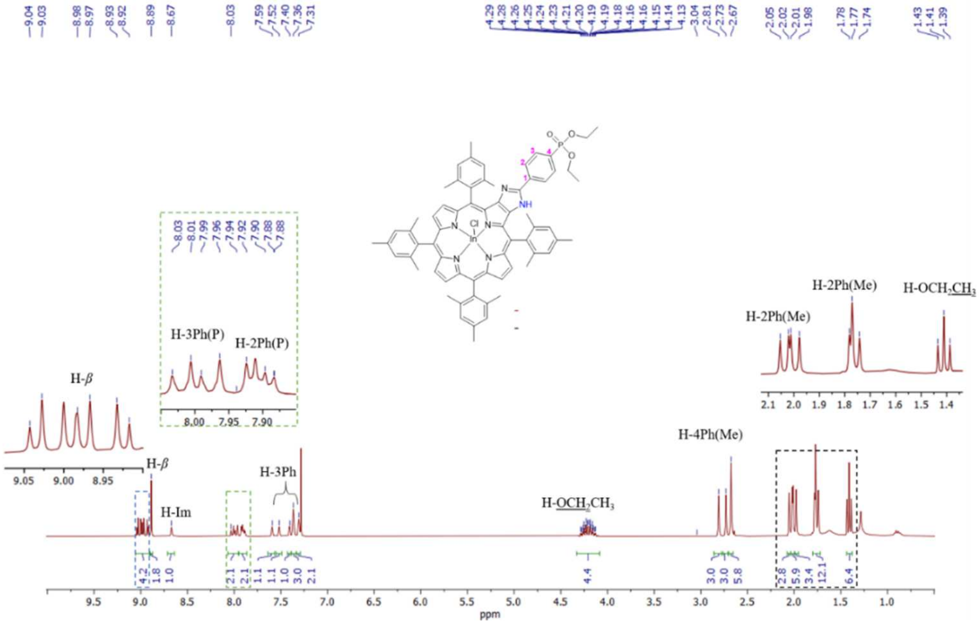

Figure S12. ^{1}H NMR spectrum of InTMPIP (300 MHz, $CDCl_3$, 298 K).

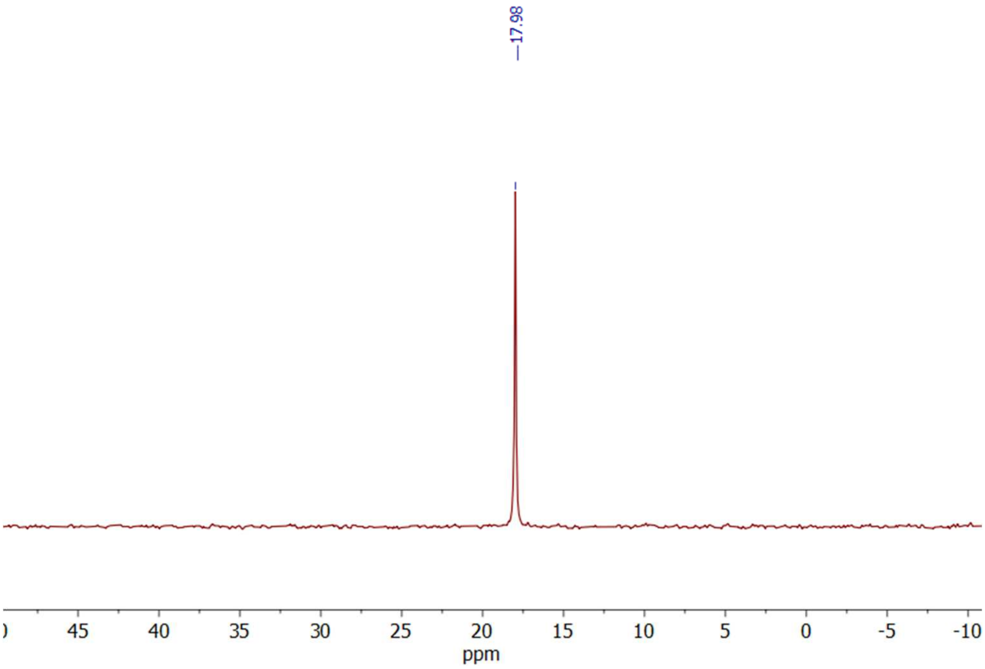

Figure S13. ^{31}P {^{1}H} NMR spectrum of InTMPIP (121 MHz, $CDCl_3$, 298 K).

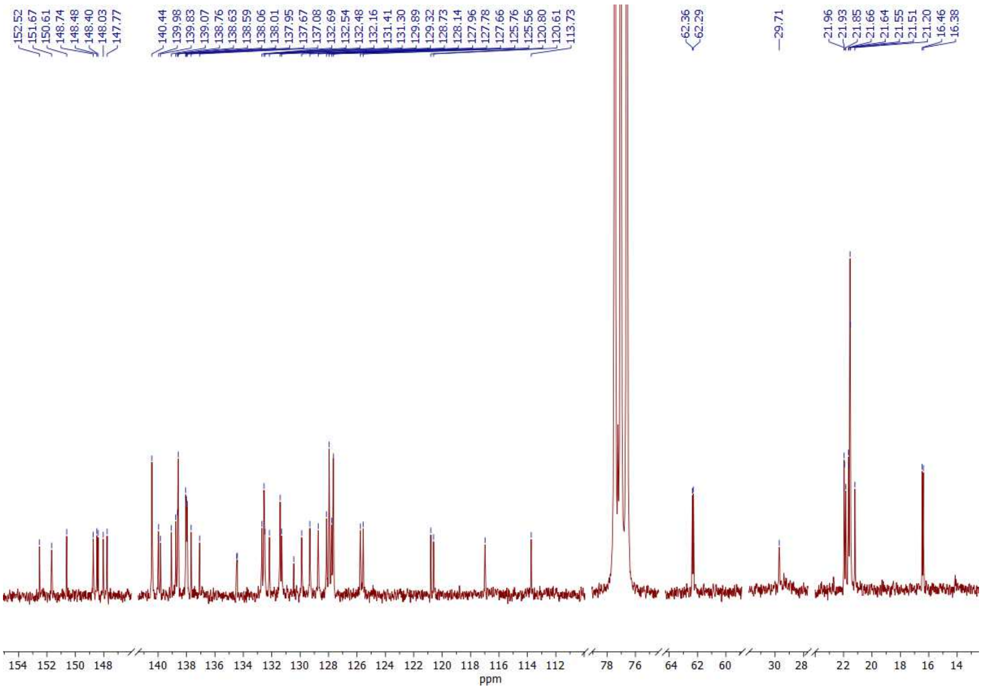

Figure S14. $^{13}C\{^1H\}$ NMR spectrum of InTMPIP (75 MHz, $CDCl_3$, 298 K).

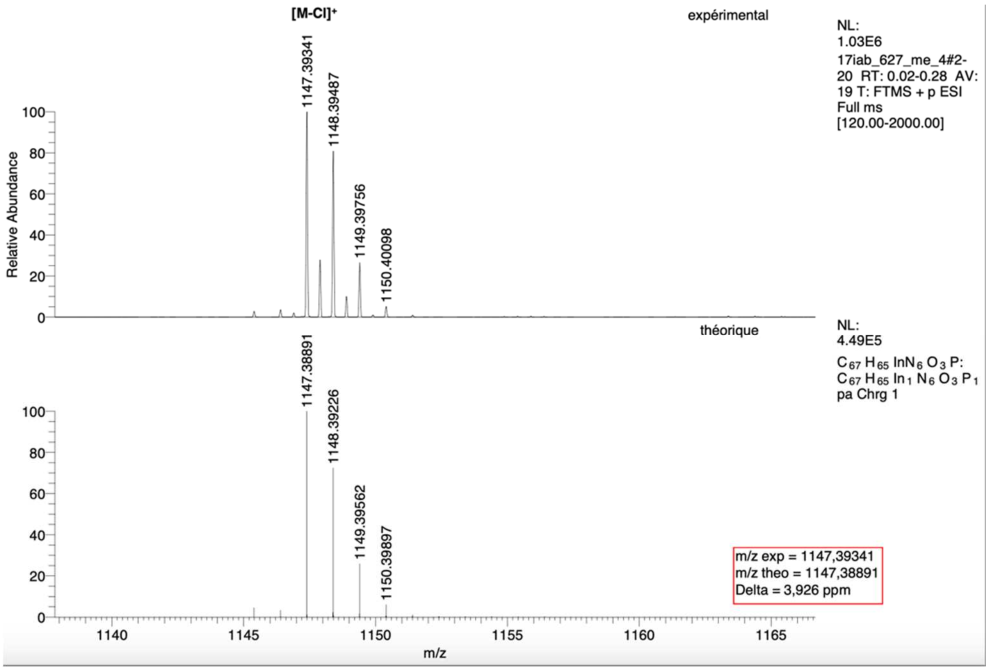

Figure S15. HRMS-ESI mass spectrum of InTMPIP.

7. NMR spectra of the reaction mixtures obtained in the photooxidation of sulfides

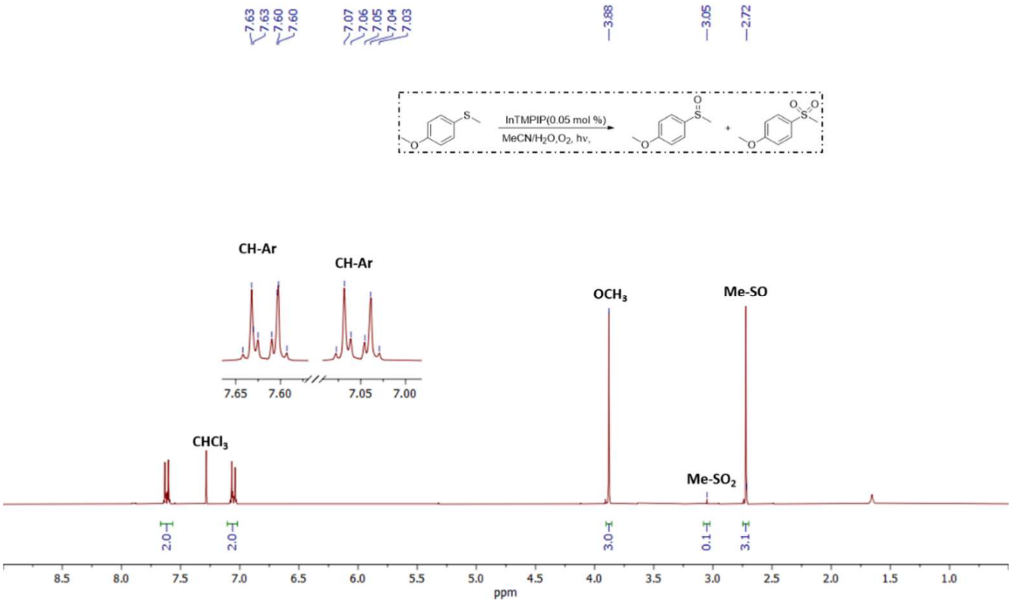

Figure S16. ^{1}H NMR spectrum (300 MHz, $CDCl_3$, 298 K) of the reaction mixture obtained from the oxidation of 4-methoxythioanisole.

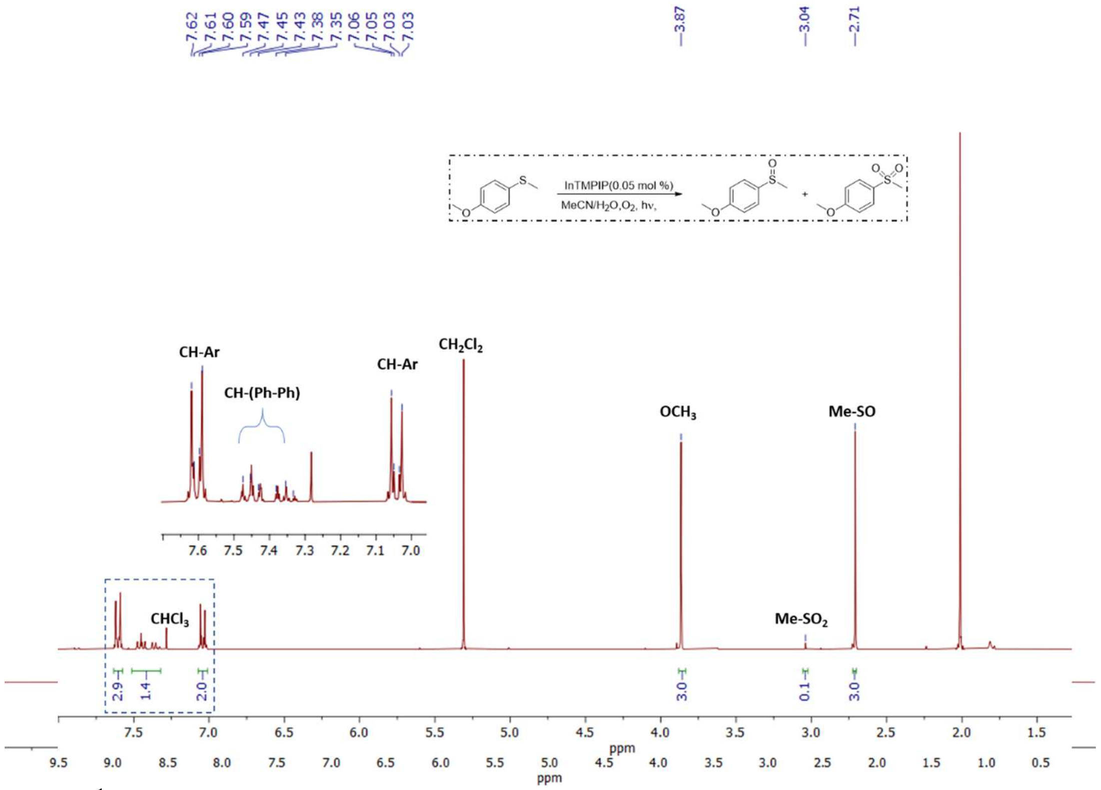

Figure S17. ^{1}H NMR spectrum (300 MHz, $CDCl_3$, 298 K) of the reaction mixture obtained from the oxidation of 4-methoxythioanisole (0.5 mmol) after the addition of 0.125 mmol of biphenyl.

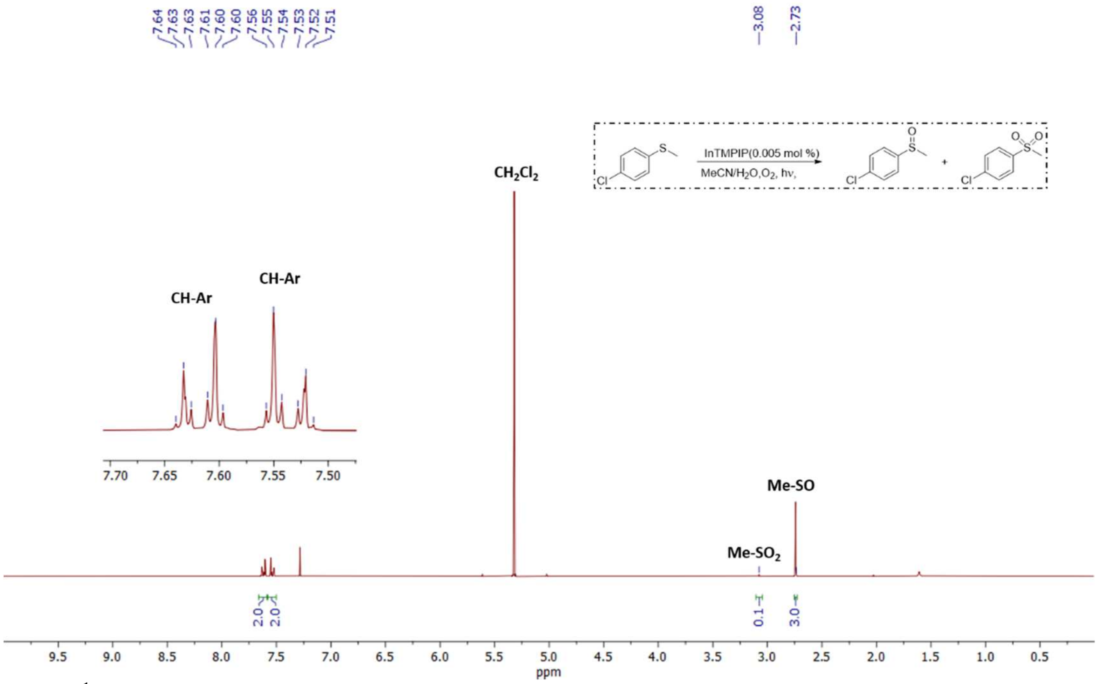

Figure S18. ¹H NMR spectrum (300 MHz, $CDCl_3$, 298 K) of the reaction mixture obtained from the oxidation of 4-chlorothioanisole.

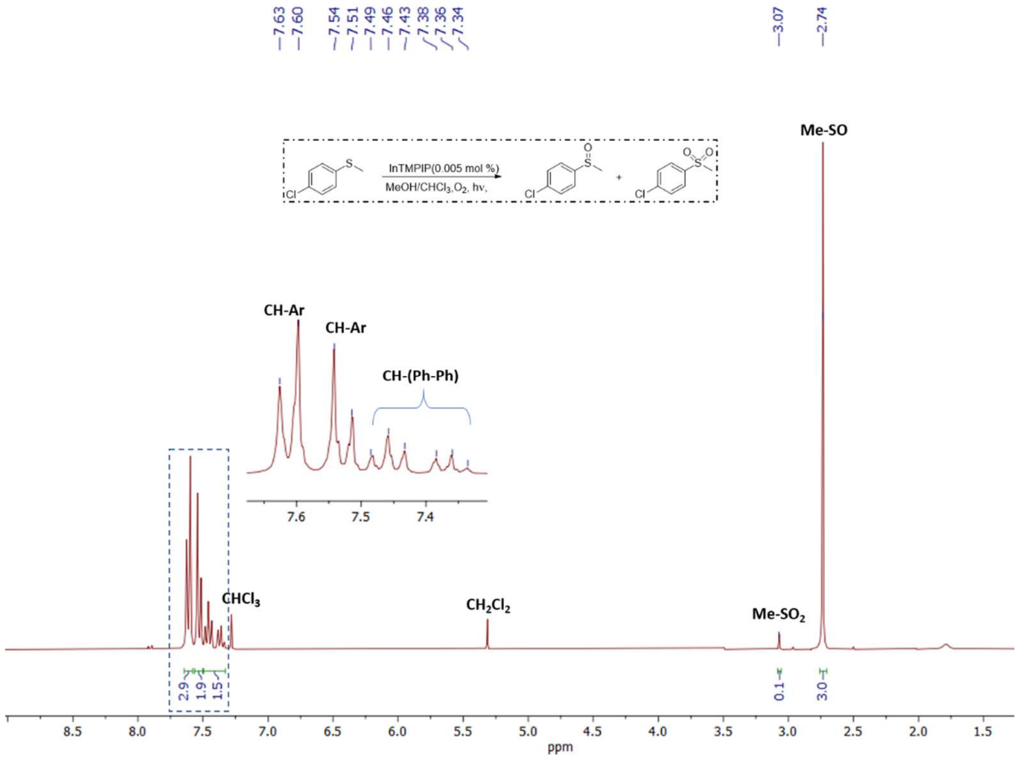

Figure S19. ¹H NMR spectrum (300 MHz, $CDCl_3$, 298 K) of the reaction mixture obtained from the oxidation of 4-chlorothioanisole (0.5 mmol) after the addition of 0.125 mmol of biphenyl.

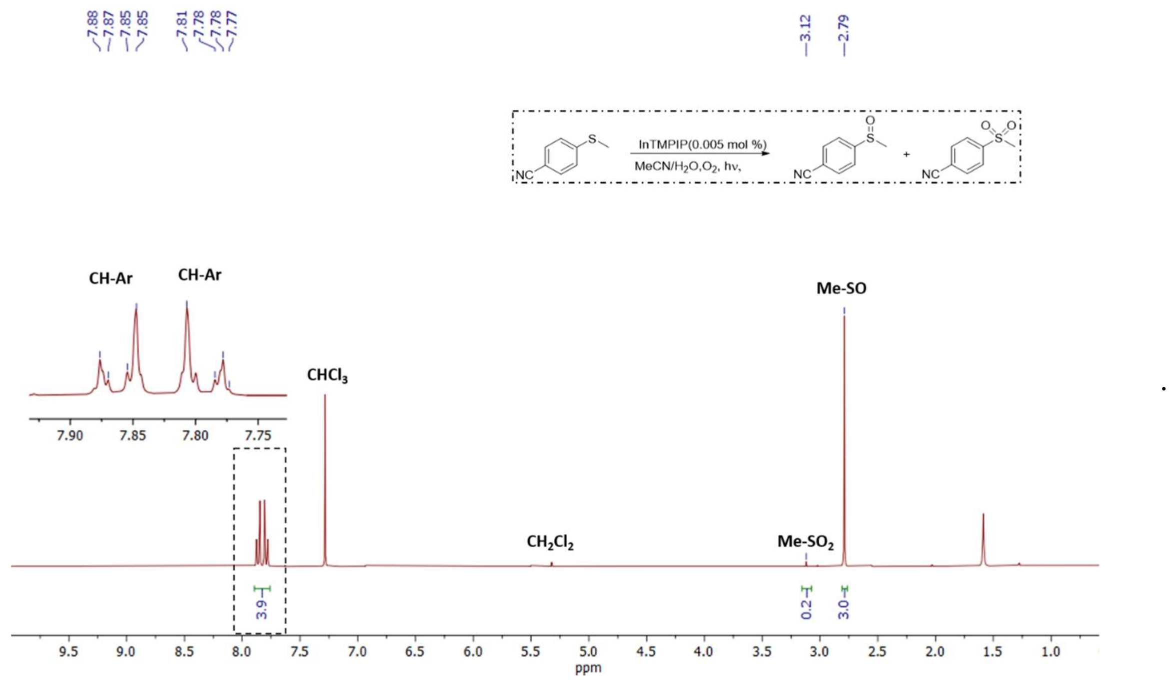

Figure S20. ^{1}H NMR spectrum (300 MHz, $CDCl_3$, 298 K) of the reaction mixture obtained from the oxidation of 4-cyanothioanisole.

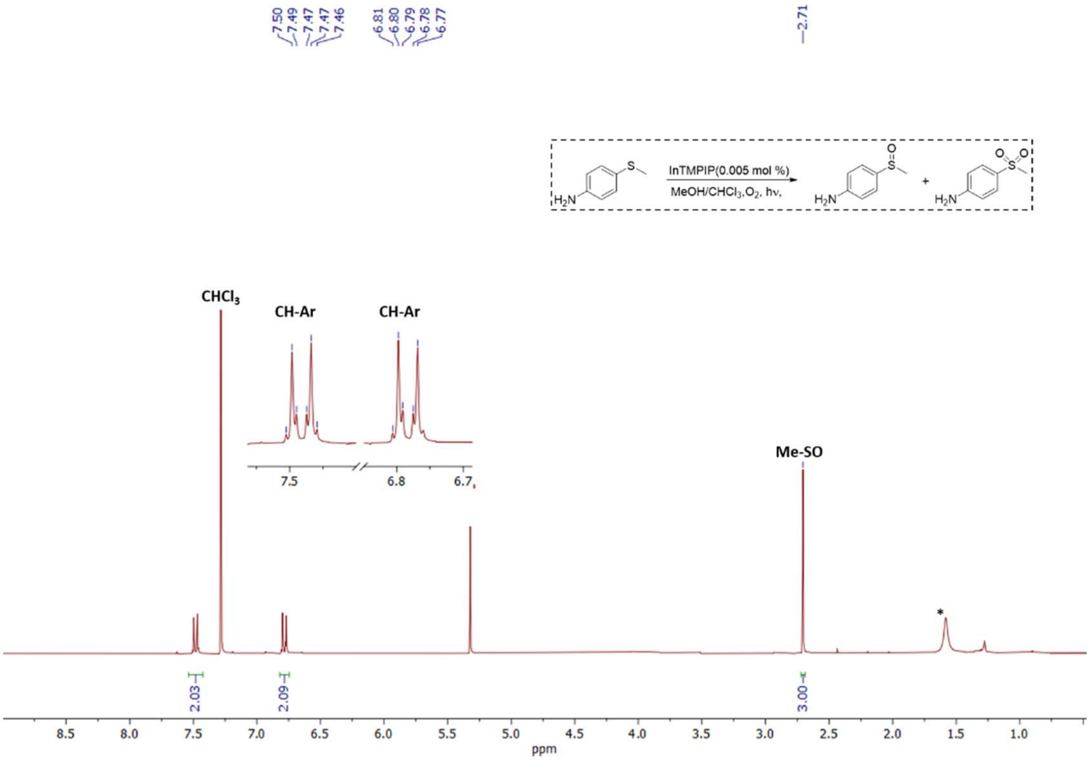

Figure S21. ^{1}H NMR spectrum (300 MHz, $CDCl_3$, 298 K) of the reaction mixture obtained from the oxidation of 4-aminothioanisole.

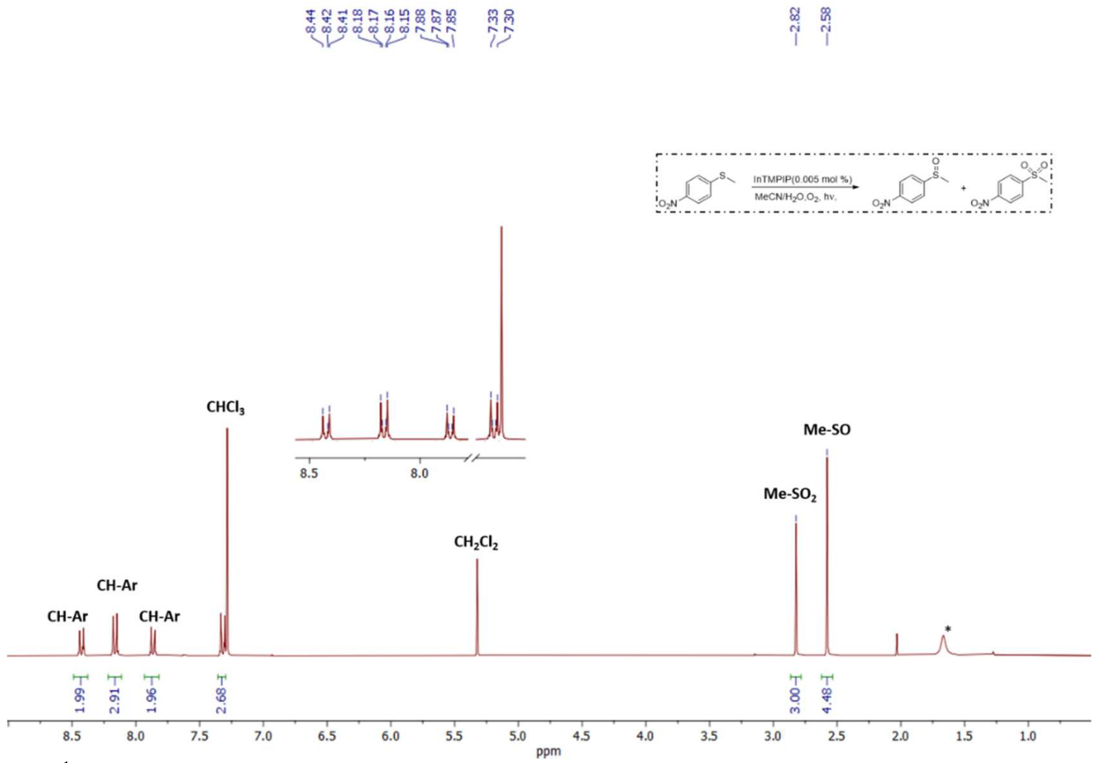

Figure S22. ^{1}H NMR spectrum (300 MHz, $CDCl_3$, 298 K) of the reaction mixture obtained from the oxidation of 4-nitrothioanisole.

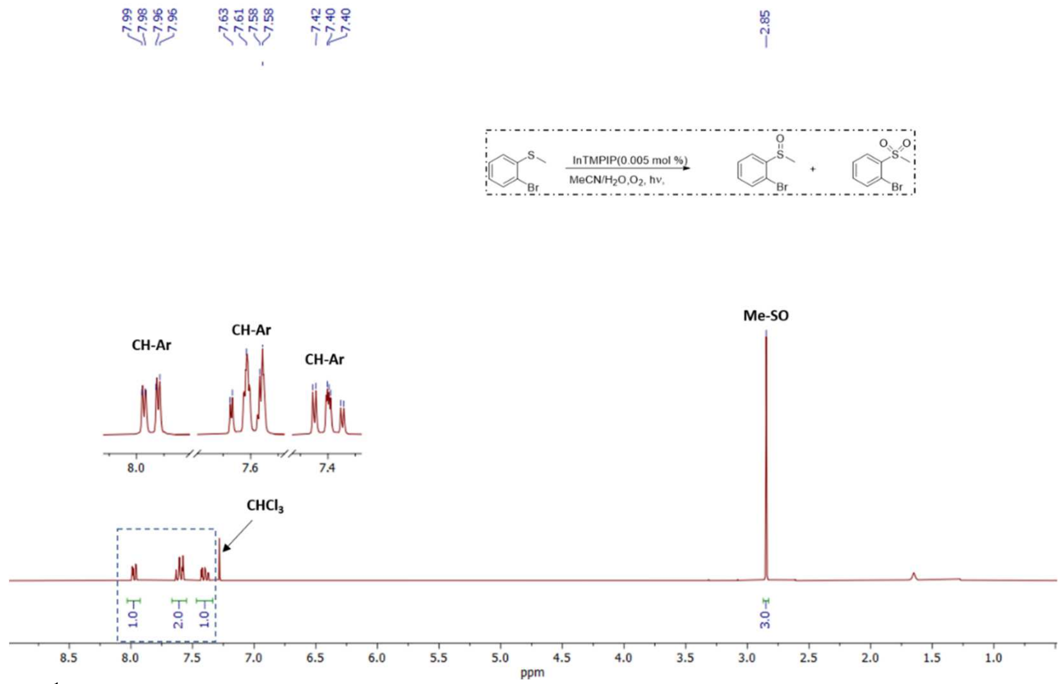

Figure S23. ^{1}H NMR spectrum (300 MHz, $CDCl_3$, 298 K) of the reaction mixture obtained from the oxidation of 2-bromothioanisole.

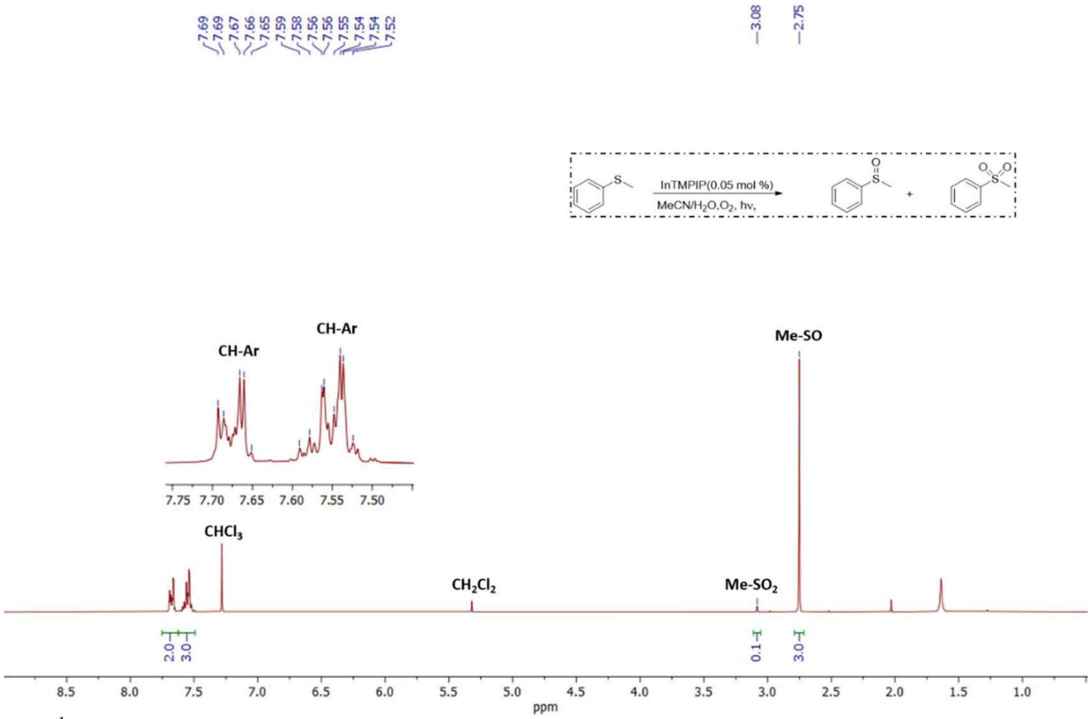

Figure S24. ^{1}H NMR spectrum (300 MHz, $CDCl_3$, 298 K) of the reaction mixture obtained from the oxidation of thioanisole.

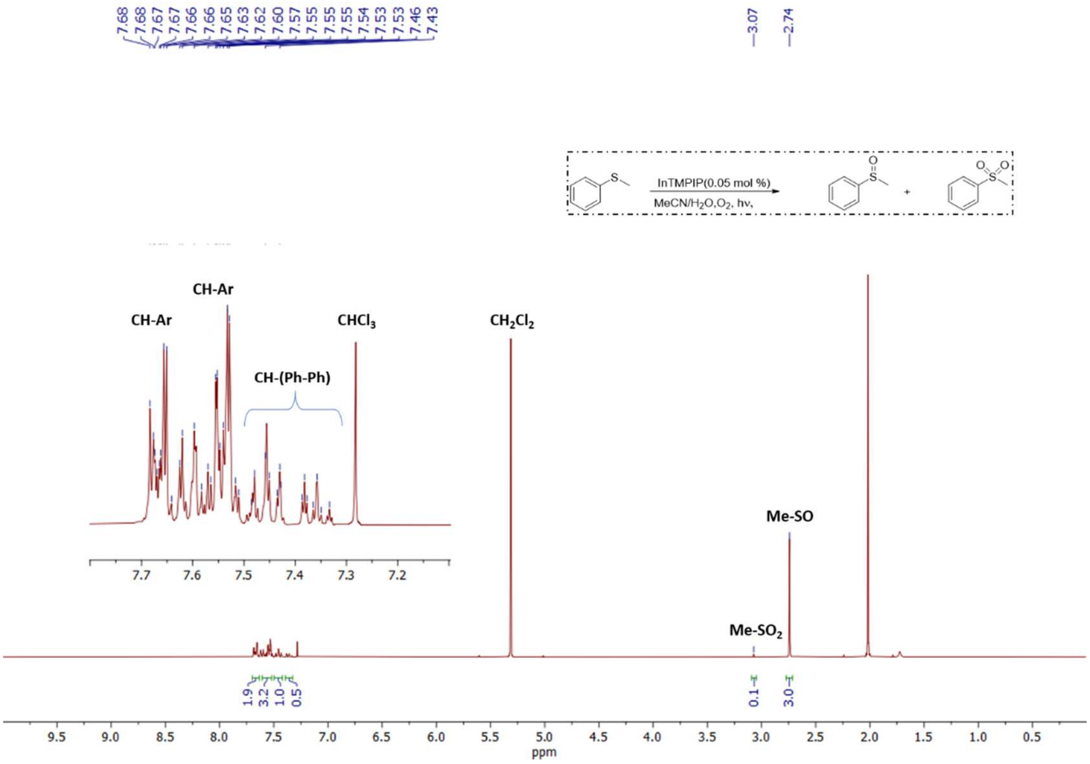

Figure S25. ^{1}H NMR spectrum (300 MHz, $CDCl_3$, 298 K) of the reaction mixture obtained from the oxidation of thioanisole (0.5 mmol) after the addition of 0.125 mmol of biphenyl.

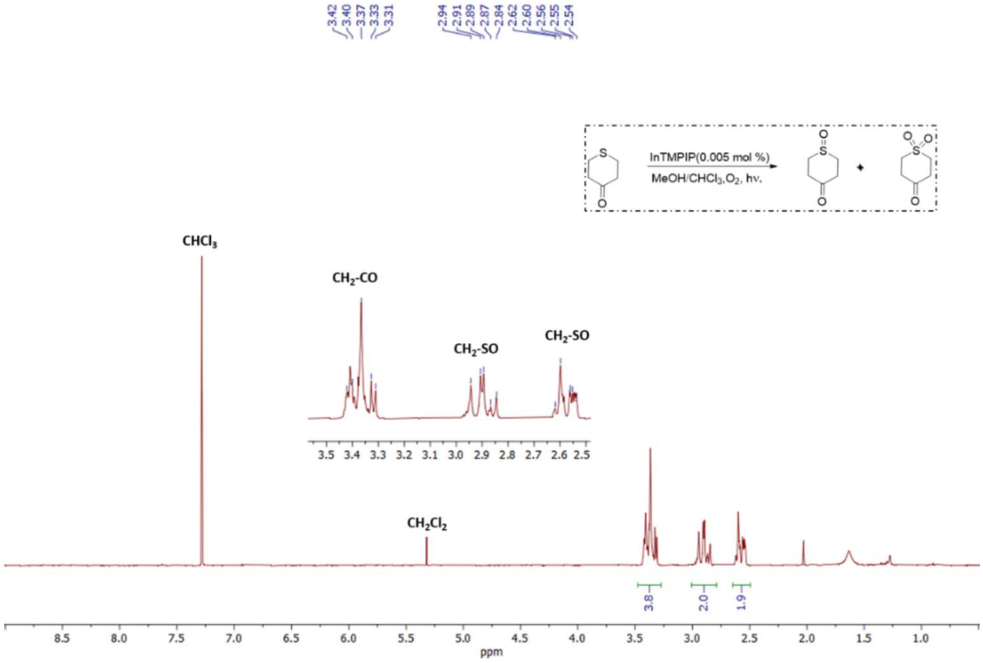

Figure S26. ^{1}H NMR spectrum (300 MHz, $CDCl_3$, 298 K) of the reaction mixture obtained from the oxidation of thian-4-one.
